# Bonding Trends in Tetravalent Th–Pu Monosalen Complexes

**DOI:** 10.1002/chem.202003241

**Published:** 2020-11-09

**Authors:** Thomas Radoske, Juliane März, Michael Patzschke, Peter Kaden, Olaf Walter, Moritz Schmidt, Thorsten Stumpf

**Affiliations:** ^1^ Institute of Resource Ecology Helmholtz-Zentrum Dresden-Rossendorf (HZDR) Bautzner Landstrasse 400 01328 Dresden Germany; ^2^ European Commission DG JRC, G.I.5 PO Box 2340 76125 Karlsruhe Germany

**Keywords:** bonding analysis, covalency, pyridine, salen, tetravalent actinide

## Abstract

The synthesis of three complex series of the form [AnCl_2_(salen)(Pyx)_2_] (H_2_salen=*N*,*N′*‐bis(salicylidene)ethylenediamine; Pyx=pyridine, 4‐methylpyridine, 3,5‐dimethylpyridine) with tetravalent early actinides (An=Th, U, Np, Pu) is reported with the goal to elucidate the affinity of these heavy elements for small neutral N‐donor molecules. Structure determination by single‐crystal XRD and characterization of bulk powders with infrared spectroscopy reveals isostructurality within each respective series and the same complex conformation in all reported structures. Although the trend of interatomic distances for An−Cl and An−N (imine nitrogen of salen or pyridyl nitrogen of Pyx) was found to reflect an ionic behavior, the trend of the An−O distances can only be described with additional covalent interactions for all elements heavier than thorium. All experimental results are supported by quantum chemical calculations, which confirm the mostly ionic character in the An−N and An−Cl bonds, as well as the highest degree of covalency of the An−O bonds. Structurally, the calculations indicate just minor electronic or steric effects of the additional Pyx substituents on the complex properties.

## Graphical abstract

**Th–Pu^IV^ salen complexes**: A series of monosalen complex with Th–Pu^IV^ has been synthesized and characterized in the solid state and with quantum chemical (QC) calculations. They reveal the salen's potential to provide a fixed binding pocket to An^IV^, allowing a facile exchange of pyridine‐based solvents in the labile positions. Differences in binding strength explain the findings: all An^IV^−N are of mostly ionic nature but An^IV^−O have a significant covalent contribution.

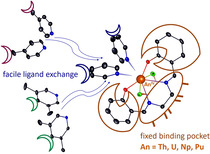

## Introduction

Prof. Karl Hensen researched and taught for several decades at the Johann Wolfgang Goethe University in Frankfurt. One of his main interests was to describe and understand the interaction of tetravalent main group elements with nitrogen heterocycles. His idea was to use these compounds to better understand the concept of covalency (also from a theoretical point of view). It is a pleasure and honor for us to take up some of the thoughts of Prof. Karl Hensen and transfer some of “his” basic main group compounds to tetravalent actinides.^[1]^

Of the wide range of accessible oxidation states within the early actinides (An) up to Am, the tetravalent one is one of the predominant oxidation states under environmental and especially under reductive conditions. In addition, the tetravalent actinides (An^IV^) are the largest series among the early An and have thus already been used to investigate bonding trends^[2]^ to give answers to questions like trends in complex stability, reactivity, or the concept of covalency.[^2g^, ^3^] Recently, also fundamental studies on actinide separation^[2d]^ or NMR parameters^[4]^ have confirmed the need of analyzing series of An^IV^ compounds. Besides the actinide center, the ligand choice with its electronic or geometric situation has a huge impact on the complex properties. Schiff bases are well‐known complexing agents for almost every oxidation state and metal ion in the periodic table. They are easily tunable regarding their steric and electronic properties and provide access to stable complexes with an impressive scope of applications, for example, catalytic activity,^[5]^ metal extraction,^[6]^ magnetism,^[7]^ or luminescence.[^7a^, ^7c^, ^8^] Schiff bases are also known to allow for partial saturation of the coordination sphere of a transition metal or An, which subsequently allows reactivity at the labile complex positions, for example, with biomolecules or for catalysis.^[9]^

In this study, we investigate solvent exchange reactions of monosalen complexes using pyridine, 4‐picoline, and 3,5‐lutidine as co‐ligands. We apply single‐crystal (SC)‐XRD data and quantum chemical (QC) calculations to reveal changes in bonding and potential covalent contributions, which can be related to the complexes’ electronic and steric properties and the influence of ligands on the labile positions.

## Results and Discussion

**Scheme 1:**
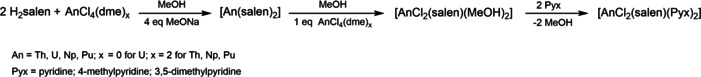
Synthesis of the An^IV^ monosalen Pyx complexes [AnCl_2_(salen)_2_(Pyx)_2_] **1**–**12**.

Three series of tetravalent actinide complexes [AnCl_2_(salen)(Pyx)_2_] (An=Th, U, Np, Pu; Pyx=pyridine (py), 4‐methylpyridine (pic), 3,5‐dimethylpyridine (lut)) were synthesized by a three‐step synthesis via [An(salen)_2_] and [AnCl_2_(salen)(MeOH)_2_] (see the Supporting Information for details) by solvent exchange reactions, always starting from [AnCl_2_(salen)(MeOH)_2_] and dissolving it in the respective pyridine‐based solvent (see Scheme 1 and the Supporting Information). After layering the complex solutions with pentane, all synthesis attempts of **1**–**12** yielded single crystals suitable for single‐crystal X‐ray diffraction.

### Structural description

Each series of An^IV^ monosalen complexes [AnCl_2_(salen)(py)_2_] (An=Th–Pu (**1**–**4**)), [AnCl_2_(salen)(pic)_2_] (An=Th–Pu (**5**–**8**)), and [AnCl_2_(salen)(lut)_2_] (An=Th–Pu (**9**–**12**)) isomorphously crystallize in a triclinic (*P* $\bar{1}$ ), orthorhombic (*Aea*2), or monoclinic (*P*2_1_/*c*) space group, respectively.

**Figure 1:**
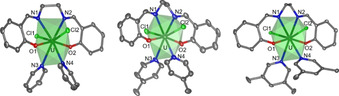
Molecular structures of [UCl_2_(salen)(Pyx)_2_] (Pyx=pyridine (**2**, left), 4‐methylpyridine (**6**, middle), and 3,5‐lutidine (**10**, right)). Ellipsoids are drawn at the 50 % probability level and hydrogen atoms are omitted for clarity. Color code: carbon (C, dark gray), oxygen (O, red), nitrogen (N, blue), chlorine (Cl, light green), uranium (U, dark green). The coordination polyhedron is displayed in a translucent green.

The isostructurality of each series was also confirmed by identical band positions in the IR spectra of the solid‐phase compounds (see the Supporting Information). As representatives for all three series, the U^IV^ complexes **2**, **6**, and **10** are shown in Figure 1.

In the three complex structures, the uranium atom is always eightfold coordinated by one tetradentate binding salen ligand, two chloro ligands, as well as two pyridine‐based solvent molecules (Pyx), analogous to the monosalen complexes of Blessing^[11]^ or Stobbe,^[12]^ resulting in a coordination environment that can be described as either distorted square antiprismatic or snub disphenoid.^[13]^

Within the U^IV^‐Pyx series **2**, **6**, and **10**, very similar distances between each coordinating O, N, or Cl atom and uranium exist, and thus the coordination environment is almost independent of the additionally coordinating solvent Pyx. In each case, the salen ligand coordinates with marginally different bond lengths U−O_salen_ or U−N_salen_ to the metal. The average bond lengths U−O_salen_ as well as U−N_salen_ are 2.176 Å or 2.581 Å, which are at the lower end of the typical range of U−O/N bonds to salen‐type ligands.[^9^, ^11^, ^12^, ^14^] U−Cl bond lengths in **2**, **6**, and **10**, on the other hand, have an average of 2.716 Å, which is at the higher end of the expected range of U^IV^−Cl bonds in salen‐type complexes.[^11^, ^12^] Interestingly, the monosalen complexes do not undergo a structural change in the solid‐state structure when substituting the solvent. In each case, the two Pyx molecules occupy identical geometrical positions within the coordination environment. Their U−N_Pyx_ bond has an average length of 2.653 Å, about 7 pm longer than the U−N_salen_ bond, indicating a weaker interaction, corroborating the fact that a facile ligand exchange takes place at those positions as demonstrated for MeOH in the Pyx complex syntheses. The small differences between the structures **2**, **6**, and **10** potentially originate from packing effects, which are, for example, influenced by structurally embedded solvent molecules, but may also point to small steric and electronic differences of the pyridine‐based solvents.

To analyze the geometric and electronic effects more deeply when substituting py (**2**) with pic (**6**) or lut (**10**), quantum chemical optimizations of the respective molecules were performed. All presented electronic structure calculations were done with the software packages Turbomole 7.3.1^[15]^ and Orca 4.2.1^[16]^ (for computational details please see the Computational Section).

All calculated structures show extremely similar coordinating bond lengths to U^IV^ compared to the SC‐XRD structures, deviating by a maximum of 2.5 pm for U−O_salen_, U−N_salen_, and U−N_Pyx_. The calculated bond lengths are minimally shorter than the experimental ones, which is most prominent for the U−Cl bonds, with differences of 2–6 pm.

**Figure 2:**
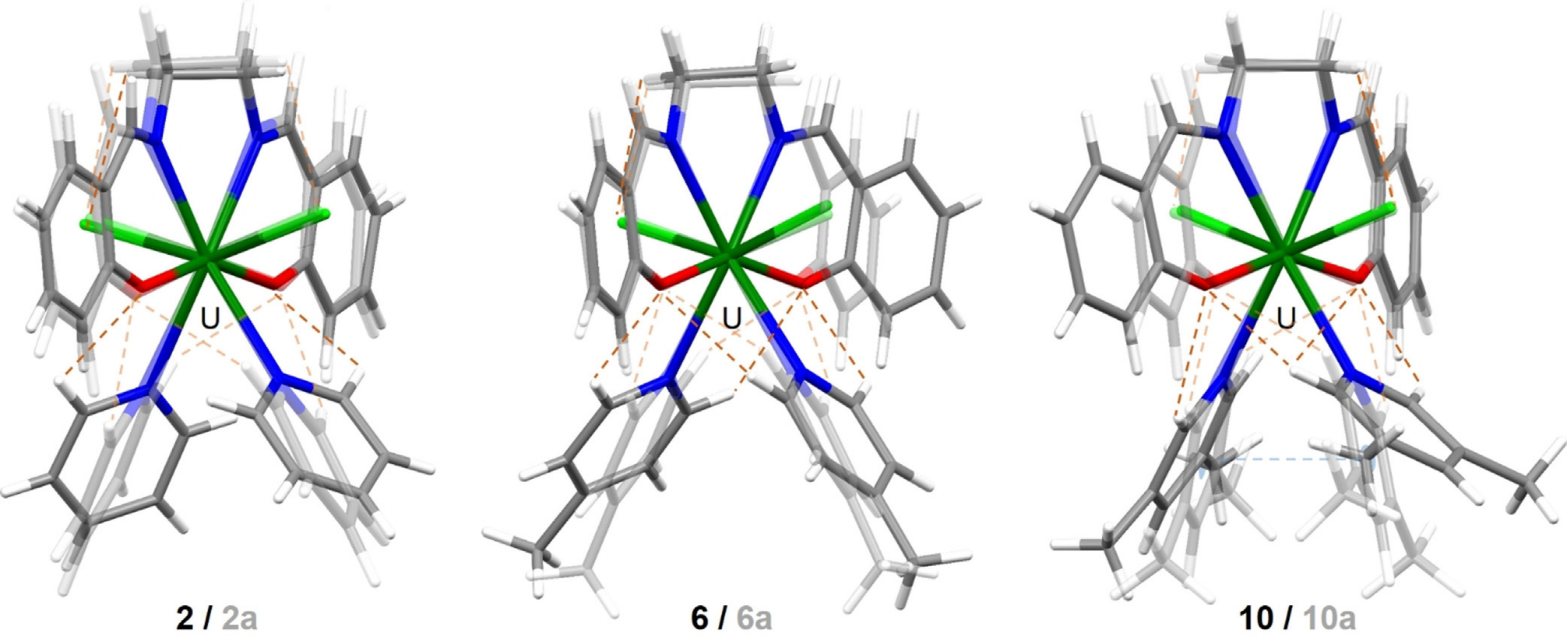
Molecular SC‐XRD structures/optimized structures (translucent) of [UCl_2_(salen)(Pyx)_2_] (Pyx=pyridine (**2/2 a**, left), 4‐methylpyridine (**6/6 a**, middle), and 3,5‐lutidine (**10/10 a**, right)). Color code of capped sticks: carbon (C, dark gray), oxygen (O, red), nitrogen (N, blue), chlorine (Cl, light green), uranium (U, dark green). The optimized structures are displayed at a 50 % translucence level. Weak interactions are drawn in dashed lines (hydrogen bonds in orange, π⋅⋅⋅π interaction in blue).

The performed calculations cannot take ordered intermolecular packing effects in crystals into account; hence, the chlorine is free to move somewhat closer to the actinide compared with the measured structures. The absence of intermolecular interactions in the calculations also explains the most significant differences between the experimentally determined and the optimized U^IV^ monosalen Pyx complexes, which is their unlike geometrical arrangement, with generally more symmetrical structures for the optimized complexes **2 a**, **6 a**, and **10 a** (see Figure 2).

The differences between experimental and calculated structures are twofold: first, the salen ligand coordinates more planarly to U^IV^ in the optimized structures, and second, the Pyx molecules are less twisted along the U‐N_Pyx_‐*para*‐C_Pyx_ axes as well as tilted against the U−N_Pyx_ bond. The latter can be expressed by the Pyx‐Pyx plane angles, which are significantly smaller (by ≈20°) in **2 a** and **6 a** compared with **2** and **6** (see Table S7 in the Supporting Information). The largest difference can be found for the lut complexes (24.7° in **10 a** vs. 83.0° in **10**, that is, 58.3°), whereas a slightly smaller difference of about 27° was found for the methyl‐substituted pyridine derivatives. The average Pyx‐Pyx plane angle in **6 a** and **10 a** is 54.3° and 81.7° in **6** or **10**. The changed arrangement of the salen ligand is likely a consequence of the changed Pyx coordination. In **2** it coordinates almost planarly to the metal center (measured by the angle of about 177° between the phenyl planes in salen), but is slightly twisted in the cases of **6** and **10** (157° and 158°, respectively). A further special feature of the coordinating 4‐methylpyridine (pic) and 3,5‐lutidine (lut) molecules in comparison to pyridine (py) is that one pic/lut in **6** or **10** always coordinates linearly to the uranium center, whereas the other one is angled by 168.6° (**6**) or 164.4° (**10**), measured by the angle *para*‐C_Pyx_‐N_Pyx_‐U. However, within the optimized complex series, in **2 a** and **6 a** py or pic each show an almost linear coordination.

This linear geometry improves the ligand metal interaction in the calculated structures; however, the An−N bond is weak and the small energy gain is easily overridden by crystal packing effects that can be of the order of 100 kJ mol^−1^ (see, for example, Schöne et al.^[17]^). Only for **10 a**, DFT finds both lut ligands angled by 141.8° or 162.1°, respectively.

A reason for the tilting of pic or lut in **6**, **10**, and **10 a** can be found in the electronic and steric changes in [AnCl_2_(salen)(Pyx)_2_] when changing the pyridine derivative. NCI plots of **2 a**, **6 a**, and **10 a** as well as the analysis of weak intermolecular interactions (see Figure 2, dashed lines) point to stronger interactions between the two lut substituents in **10 a** in the form of π⋅⋅⋅π stacking compared with py or pic in **2 a** or **6 a**, potentially stabilizing the tilted lut coordination. Furthermore, the sheer number of formed hydrogen bonds C_Py,*ortho*_−H_Py,*ortho*_⋅⋅⋅O_salen_ and C_salen_−H_salen_⋅⋅⋅Cl bonds is higher in the more symmetric optimized structures compared with the radiographically determined ones and might serve as an explanation for the structural differences (see orange dashed lines in Figure 2 and Supporting Information Tables S2–S4 for hydrogen bond details). To prove the impact of electronic and steric changes within the Pyx series on the binding strength to U^IV^, natural bond orbital (NBO) analyses were performed. Whereas no atom charge differences of the binding O_salen_, N_salen_, or Cl atoms can be found for py, pic, or lut, slightly more negatively charged N_Pyx_ occur at N_pic_ than N_py_ or N_lut_. These differences are already present in the free form of the ligands and reflect the difference in the substitution pattern for the three Pyx ligands. The observed charge differences do not affect the binding affinity of the U−N_Pyx_ bonds, as the calculated QTAIM metrics show almost identical values for all three Pyx substituents (see Table S14 in the Supporting Information). This means that either the methyl groups induce only marginal differences in the electronic situation of the complex, or any induced effects such as the tilting of pic and lut, the weak intramolecular interactions, or electron‐donating effects of the methyl substituents, offset in such a way the aggregate effect is negligible. Either way, additional methyl groups somewhat unexpectedly neither affect the binding of the Pyx substituents to U^IV^, nor the binding strength of the salen ligand to the same metal center.

The intermolecular packing of **2**, **6**, and **10** is dominated by an extended hydrogen‐bond network between the neighboring complex molecules as well as embedded solvent molecules of pyridine in **2** as well as 3,5‐lutidine in **10** in the form of C−H⋅⋅⋅π and C−H⋅⋅⋅Cl interactions. In addition, π⋅⋅⋅π interactions occur, originating from the coordinating Pyx molecules to embedded pyridine in **2** and to the salen ligand in **6**.

We also want to address the question of whether there is an energetic reason for the specific positioning of the Pyx ligands relative to Cl and salen observed for all complexes. Principally, this question can be answered by comparison of the solid‐state structures with those in solution, for example, by NMR spectroscopy. As representatives of the [AnCl_2_(salen)(Pyx)_2_] series, NMR spectra of [AnCl_2_(salen)(py)_2_] (Th, U) were collected in pyridine‐d_5_, revealing a 1:1 complex stoichiometry with respect to salen in solution with enormous chemical shifts in the case of the paramagnetic U^IV^ complex, that is, a range of −55 to +80 ppm in the ^1^H NMR spectrum. Unfortunately, a quick exchange of py and py‐d_5_ prohibits further characterization in solution and thus no concrete information about solution species is available.

Instead, we again resort to quantum chemical calculations on all possible alternative conformations for the chloro or pic ligands of [AnCl_2_(salen)(pic)_2_] complexes to determine their relative overall free energy. The results show that the structures derived from SC‐XRD are energetically favored by about 10–40 kJ mol^−1^ towards conformational changes, for all used tetravalent actinides, Th, Pa, U, Np, or Pu. In all cases, a single twist of one chlorine versus pic has a lower energy than the complete positional exchange (see Figures S22 and S23 in the Supporting Information).

**Figure 3:**
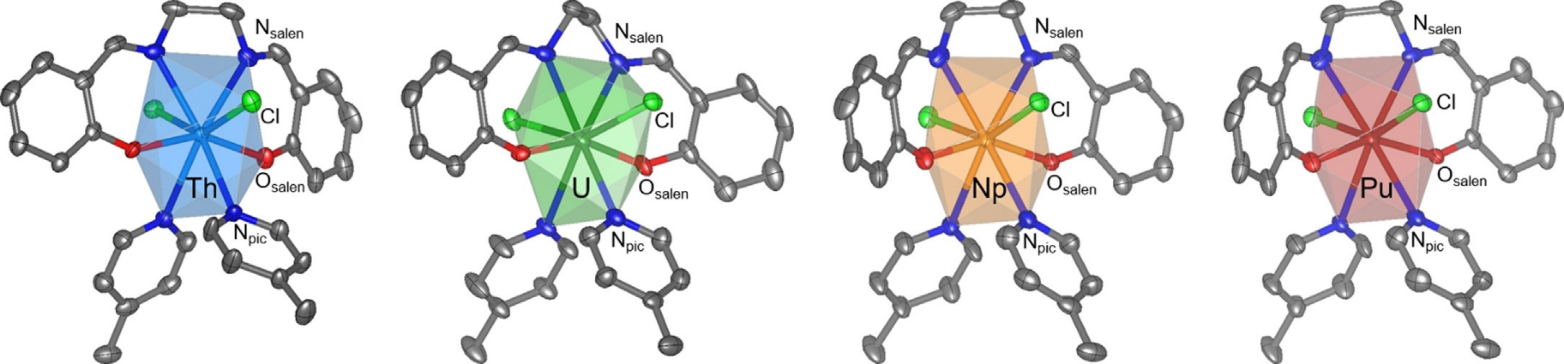
Molecular structures of [AnCl_2_(salen)(Pic)_2_] (An=Th, U, Np, Pu (from left to right)). Ellipsoids are drawn at the 50 % probability level and hydrogen atoms are omitted for clarity. Color code: carbon (C, dark gray), oxygen (O, red), nitrogen (N, dark blue), chlorine (Cl, light green), thorium (Th, light blue), uranium (U, dark green), neptunium (Np, orange), plutonium (Pu, red). The coordination polyhedra are displayed in the translucent color of the respective An.

As we established only minor effects of the labile Pyx ligands on the overall structure of the complexes, the complexes [AnCl_2_(salen)(pic)_2_] (An=Th–Pu (**5**–**8**)) were chosen as representatives (see Figure 3) for the three series to analyze bonding trends, for example, related to covalency, over the early actinide series. This includes structure optimizations and bonding analyses for the Th^IV^–Pu^IV^ complexes **5 a**–**8 a** as well as for the experimentally inaccessible Pa^IV^ complex.

While traversing the tetravalent early actinide complex series **5**–**8**, the complex conformation is preserved and thus is analogous to the U^IV^ complex **6**, which is described above. The optimized structures **5 a**–**8 a** are again slightly more symmetric compared with the SC‐XRD structures and show almost exactly the same coordinating bond lengths with deviations of only about 2 pm. Most pronounced differences again occur for the An−Cl bond lengths, which are about 4 pm shorter for the calculated structures, pointing to stronger actinide chlorine interactions for isolated gas‐phase molecules. In general, coordinating bond lengths decrease from Th^IV^ to Pu^IV^ in agreement with the decreasing cation size.

**Figure 4:**
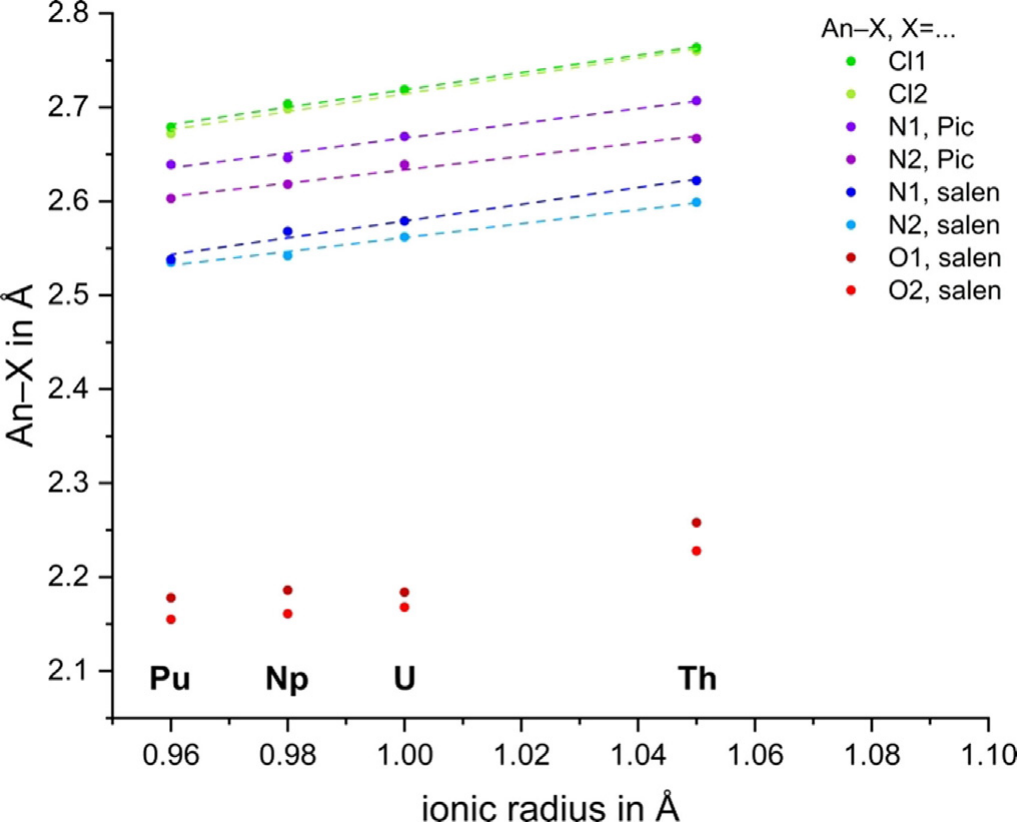
Plot of experimentally determined coordinating bond lengths in [AnCl_2_(salen)(Pic)_2_] versus the Shannon ionic radii for coordination number (CN)=8^[18]^ and the respective linear fits (dashed lines) for An−Cl and An−N.

Figure 4 shows the trends of coordinating bond lengths plotted against the ionic radii^[18]^ of the tetravalent actinides with coordination number eight (a detailed plot including the calculated bond lengths is given in Figure S13 in the Supporting Information). This graph shows that An−Cl and An−N_Pyx/salen_ bond lengths follow the decrease of the ionic radius nearly linearly. Between the two nitrogen donors N_salen_ and N_Pyx_, it is clear that the N_salen_ atoms bind closer to An^IV^ than the Pyx solvents. The Pyx solvents also bind more asymmetrically than the N_salen_ atoms, as indicated by the larger difference in An−N1/N2_Pyx_.

By using the calculated QTAIM metrics, we can relate those structural features to bond properties. We are here looking at the Delocalization Index (DI), the electron density at the bond critical point (*ρ*) and at the energy density (*H*). For predominantly covalent interactions, we would expect values of *ρ*>0.2. Values below 0.1 are indicative of closed shell or weak interactions. For such cases, it is beneficial to look at the energy density. For covalent bonds it should be negative, the size of the value indicating the strength of the covalent interaction.^[19]^

We always find two marginally different values for An−N_salen_ and An−N_Pyx_ for all three metrics considered, confirming that the An−N_salen_ bond is slightly stronger than An−N_Pyx_ (e.g., ΔDI=0.04). The electron density at the bond critical point (BCP) for the An−N interactions is around 0.05 for An−N_salen_ and 0.045 for An−N_pyx_ with slight variations over the An series. These values are low, but the energy density is negative at the BCP, indicating a weak covalent interaction, probably driven by the σ‐donor capabilities of the nitrogen.

Compared with the An−N bonds having an average DI of 0.28, the An−Cl bonds have twice that value, 0.56, pointing to substantially stronger interactions. Keeping in mind that DFT slightly underestimates the An−Cl bond lengths, this average DI is likely overestimated. Nonetheless, the large DI is interesting and might be indicative of a degree of covalency in a bond typically assumed to be entirely ionic.

The electron density at the BCP is larger than for the An−N bonds with values around 0.06 and the energy density is more negative by a factor of two compared with the An−N bonds. In passing, we note that Kerridge et al.^[20]^ found values of *ρ* of up to 0.16 for pure UX systems. Although we therefore have evidence for a covalent part in the An−Cl interaction, it is only weak and the bond is predominantly ionic. This is supported by the strict linear decrease of the bond lengths with decreasing ionic radius, which suggests an ionic behavior. This trend stems from two factors: on the one hand, the energy driven covalency effect leads to an increased covalent bond in the early An series until Np/Pu owing to enhanced metal and ligand orbital overlap, and reflect, on the other hand, the more ionic bond character the heavier the An becomes.^[2g]^

The highest DI for any bond in the [AnCl_2_(salen)(pic)_2_] series is found for the An−O bonds, with an average DI(An−O) of 0.60. For comparison, our DI of 0.6 and value of *ρ* of around 0.1 is slightly larger than values reported for Ce and U double bonds to C,^[21]^ but smaller than the record value of 0.2 reported for Ce/U double bonds to O.^[22]^ From this we conclude that the An−O bond shows a relatively high degree of covalency, which is further confirmed by comparing the An−O bond lengths over the Th–Pu series with the ions’ radii (Figure 4). In this case, the bond length does not follow the trend of ionic radii but accurately reflects the trend of covalent radii instead.^[23]^ This example nicely describes that softer donors according to the hard soft acid base (HSAB) concept do not necessarily need to show a higher degree of covalency in their bonds to tetravalent early actinides.^[3c]^

Within the Th–Pu series of [AnCl_2_(salen)(pic)_2_], the DIs increase, with the clearest trend seen in the An−O bond. For this bond, the values of *ρ* and *H* seem to indicate that a maximum of covalency is reached for uranium (see Figure S14 in the Supporting Information). All other series of DIs show an increase from Th to Pa and just minimally increase thereafter. Similar trends in the DIs have been seen, for example, in the An(COT)_2_ series.^[3a]^ The other QTAIM metrics behave less linearly. This is due to the low covalency in these interactions. Nevertheless, the 5f electrons seem to play a role in bond strengthening with the highest DIs found for the 5f^4^ Pu^IV^ complexes. But even though the DIs are highest for the Pu^IV^ complex, the asymmetric Pu‐donor bond lengths as well as differences of DI for each pair of coordinating Cl, O, N_salen_, or N_Pyx_ bonds point to non‐ideal bonding situations. It is conceivable that Pu^IV^ is already slightly too small to fit into the salen pocket, or that the non‐ideality is a result of the onset of steric crowding.

## Conclusion

Three series of tetravalent actinide complexes [AnCl_2_(salen)(Pyx)_2_] (An=Th, U, Np, Pu; Pyx=pyridine (py), 4‐methylpyridine (pic), 3,5‐dimethylpyridine (lut)) **1**–**12** have been synthesized based on solvent exchange reactions, to study the electronic and steric effects of the pyridine‐based solvent. Within the U^IV^ complex series of the three different Pyx solvents, the coordination configuration is preserved. SC‐XRD structures as well as quantum chemical calculations showed that additional methyl groups on the Pyx substituent do not affect the binding strength of Pyx or of salen in the same complex. The facile exchange of Pyx substituents in [AnCl_2_(salen)(Pyx)_2_] indicates that the Pyx molecules occupy labile coordinating positions and thus have only a minor influence on the complex conformation. The labile coordination, however, indicates that these sites will be accessible for subsequent reactions. An analysis of the coordinating bonds by SC‐XRD and QC calculations further confirmed that the complex stability is mainly determined by the two chloro ligands and the salen oxygen atoms. Both the An−O and, to a lesser degree, the An−Cl bonds exhibit signs of shared electron density, and thus covalent character, clearly exceeding that of the bonds to the softer N‐donor atoms.

## Experimental Section

**Caution**! ^232^Th, ^235^U, and ^238^U are long‐lived α‐emitters with half‐lives of 1.41×10^10^, 7.04×10^8^, and 4.47×10^9^ years, respectively. ^237^Np and ^242^Pu are highly radioactive α‐emitters with half‐lives of 2.14×10^6^ and 3.76×10^5^ years, respectively. These radionuclides are also chemically toxic. Handling these radionuclides involves a serious risk to human health. Therefore, special precautions with appropriate lab equipment and facilities dedicated to radiation protection are required for handling these radioactive materials.

### Synthesis of compounds

All the synthetic work in this study was performed in an oxygen‐ and water‐free glovebox filled with nitrogen gas. The actinide starting compounds UCl_4_ and AnCl_4_(dme)_2_ (An=Th, Np, Pu, dme=dimethoxyethane) were prepared by following literature procedures.[^24^, ^25^, ^26^, ^27^] Solvents, except methanol and commercial solvents (Sigma–Aldrich, now Merck) with sufficient purity, were purified by heating at reflux with lithium aluminum hydride. All the solvents used in this study were distilled and stored over activated molecular sieves (3 Å) prior to use. The synthesis and characterization of the starting compounds [An(salen)_2_] and [AnCl_2_salen(MeOH)_2_] is described in the Supporting Information. The general synthesis of the actinide series of compounds **1**–**12** was as follows: the respective actinide complex with methanol on its labile coordination sites (i.e., [AnCl_2_salen(MeOH)_2_] with An=Th, U, Np, Pu) was dissolved in pyridine (**1**–**4**), 4‐picoline (**5**–**8**), or 3,5‐lutidine (**9**–**12**). The solution was evaporated to yield the bulk powder (only Th and U compounds) or layered with pentane to yield single crystals. Evaporation for the bulk powder synthesis was accelerated by application of a weak vacuum. Please note that SC‐XRD analysis revealed one pyridine or one 4‐picoline molecule per complex molecule **1**–**4** or **5**–**8**, respectively, as well as half a 3,5‐lutidine molecule per complex molecule **9**–**12**. Additionally, please note that it was not possible to obtain pure elemental analysis data for all complexes containing labile coordinated solvent molecules. For example, remaining solvent 4‐picoline and 3,5‐lutidine could not been removed completely from the bulk powders owing to their high boiling points (for details, please see the Supporting Information).

### Single‐crystal X‐ray diffraction (SC‐XRD)

Single‐crystal X‐ray diffraction measurements were performed at 100 K with a Bruker D8 VENTURE diffractometer equipped with an IμS microfocus X‐ray source (Mo_Kα_ radiation, *λ*=0.71073 Å), a PHOTON 100 CMOS detector, and an Oxford Cryostream cryostat. Crystals suitable for diffraction measurements were selected with an optical microscope equipped with polarization filters, transferred into mineral oil, and mounted onto a polymer loop MicroMount supplied from MiTeGen, USA. Narrow frame data collected with generic φ‐ and ω‐scans were treated with the Bruker APEX3 program suite and integrated with the SAINT software package.^[28]^ Absorption correction on the collected reflections was initially performed numerically based on the actual size and shape of the measured crystal, and was further performed empirically by the multi‐scan method (SADABS).^[29]^ Structure determination and refinement were performed with full‐matrix least‐squares data on *F* ^2^ by direct methods with the Olex2 program with SHELX program.^[30]^ Apart from the disordered structure, all non‐hydrogen atoms were refined anisotropically. Hydrogen atoms were initially placed at calculated positions and allowed to ride around their parent atoms upon refinement. In the crystal packing of the complexes [AnCl_2_(salen)(pic)_2_], solvent accessible voids are present. Diffuse electron density within the voids was treated with the program PLATON/SQUEEZE.^[31]^

### Powder X‐ray diffraction (PXRD)

Powder X‐ray diffraction profiles were collected at ambient conditions with a Rigaku MiniFlex 600 equipped with a Cu_Kα_ radiation source (*λ*=1.54184 Å) and a D/teX Ultra Si strip detector in the Bragg–Bretano geometry (*θ*−2*θ* mode). Collected data were treated and analyzed with the software PDXL.6.

### Infrared (IR) spectroscopy

The IR spectra of all described compounds were measured with an Agilent Cary 630 FTIR spectrometer using a single‐reflection attenuated total reflection (ATR) with a diamond crystal sample holder. In the case of transuranium compounds, the respective complex solution was dropwise evaporated on the diamond crystal to yield IR spectra with minimal waste generation.

### Elemental analysis

The contents of H, C, and N in the Th^IV^ and U^IV^ compounds **1**, **2**, **5**, **6**, **9**, and **10** were determined with a vario MICRO cube (Elementar) with a helium gas flow.

### NMR spectroscopy

NMR spectra were recorded with a Varian Inova 400 spectrometer with a ^1^H frequency of 399.89 MHz and a ^13^C frequency of 100.56 MHz. All spectra were recorded by using a Varian AutoX ID probe head with *z* gradient. Deuterated solvents were purchased from Deutero GmbH and dried over a potassium mirror prior to use.

### Computational Section

All presented electronic structure calculations were done with the software packages Turbomole 7.3.1^[14]^ and Orca 4.2.1.^[15]^ First preoptimizations were performed with the DFT functional PBE0^[32]^ and a def2‐SVP^[33]^ basis set for all atoms. The PBE0 functional was chosen as it is known to perform well for all elements in the periodic table.^[34]^ Furthermore, PBE0 gives reliable electron densities,^[35]^ which are of course important for the QTAIM analysis. For the actinides, the basis set includes an effective core potential (ECP) to include scalar relativistic effects, the used small core ECPs have 60 electrons in the core.^[36]^ The preoptimized structures were then further optimized with a larger def2‐TZVPP basis set. For all structures, vibrational frequencies were calculated to ensure that minima on the potential hyper surface were located. All DFT calculations used Grimme's D3 correction to include dispersion effects^[37]^ and used the COSMO^[38]^ approach with the dielectric constant set to infinity to approximate packing effects in an averaging manner. All final structures were then subjected to an NBO analysis^[39]^ as implemented in the Turbomole package. The Gibbs free energy values for the structural isomers were determined by using partition sums in a harmonic approximation and ideal gas approximation. To perform QTAIM^[40]^ analyses of all structures, all‐electron calculations were done with the Orca package by using a SARC basis set of TZVPP quality for the actinides^[41]^ and a def2‐TZVPP basis set for all other atoms. Scalar relativistic effects were taken into account by the DKH approximation.^[42]^ From the results of these calculations, input files for the QTAIM analysis were obtained with the molden2aim program.^[43]^ The QTAIM analysis and visualization of its results were done with the AIMALL package (version 19.10.12).^[44]^

## Conflict of interest

The authors declare no conflict of interest.

## Supporting information

As a service to our authors and readers, this journal provides supporting information supplied by the authors. Such materials are peer reviewed and may be re‐organized for online delivery, but are not copy‐edited or typeset. Technical support issues arising from supporting information (other than missing files) should be addressed to the authors.

Supplementary
