# Supplementary material for: Bonding Trends in Tetravalent Th–Pu Monosalen Complexes
Source: Chemistry. 2020 Nov 9;26(70):16853–9. doi: 10.1002/chem.202003241 (PMC7984231; doi:10.1002/chem.202003241)
Supplement: Supplementary file 1 — Supplementary [file CHEM-26-16853-s001.pdf]

Chemistry–A European Journal

Supporting Information

Bonding Trends in Tetravalent Th–Pu Monosalen Complexes

Thomas Radoske,[a] Juliane März,*[a] Michael Patzschke,[a] Peter Kaden,[a] Olaf Walter,[b] Moritz Schmidt,[a] and Thorsten Stumpf[a]

Supporting Information

Bonding Trends in Series of Tetravalent Th-Pu Monosalen Complexes

T. Radoske,[a] J. März,*[a] M. Patzschke,[a] P. Kaden,[a] O. Walter,[b] M. Schmidt,[a] and T. Stumpf [a]

This article is dedicated to Prof. Karl Hensen on the occasion of his 85th birthday.

[a] T. Radoske, Dr. J. März, Dr. M. Patzschke, Dr. P. Kaden, PD Dr. habil. M. Schmidt, Prof. Dr. T. Stumpf
Helmholtz-Zentrum Dresden-Rossendorf (HZDR)
Institute of Resource Ecology
Bautzner Landstraße 400, 01328 Dresden, Germany
E-mail: j.maerz@hzdr.de

[b] O. Walter
European Commission
DG JRC, G.I.5,
PO Box 2340, D-76125 Karlsruhe, Germany

Table of contents

Experimental details 3

Elemental Analysis, IR bands and NMR shifts 3

Crystal structure 10

Structure parameters derived from SC-XRD and QC calculations....................................16

Hydrogen bonds and π interactions..16

Coordinating bond lengths from SC-XRD and QC calculations......................................17

Results from quantum chemical calculations 19

Geometry optimisations in comparison with SC-XRD results 19

NBO analysis 20

Delocalisation indices (DI's) 21

Structure optimization after conformational changes of [$ThCl_2$(salen)(pic)$_2$] 24

IR data 25

Powder XDR patterns of bulk powders. 29

NMR spectra 31

Crystallographic data...39

Atomic coordinates x y z for optimised structures 42

Experimental details

The tetravalent actinide complexes **1**-**12** were prepared via a 3-step synthesis starting with the bissalen complexes [An(salen)$_2$] (An = Th, U, Np, Pu) (see Figure S1).

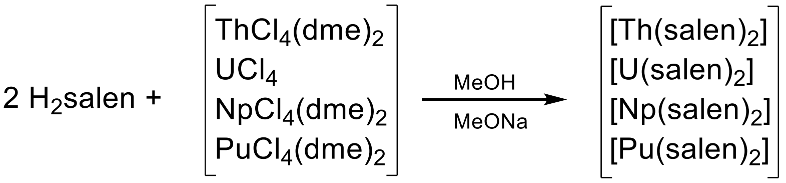

Figure S1. Synthesis route for [An^{IV}(salen)$_2$] (An(IV)=Th, U, Np, Pu)

In a second step, monosalen MeOH complexes of An(IV) have been prepared by addition of one equivalent of the metal salt to its bissalen complex (see figure S2).

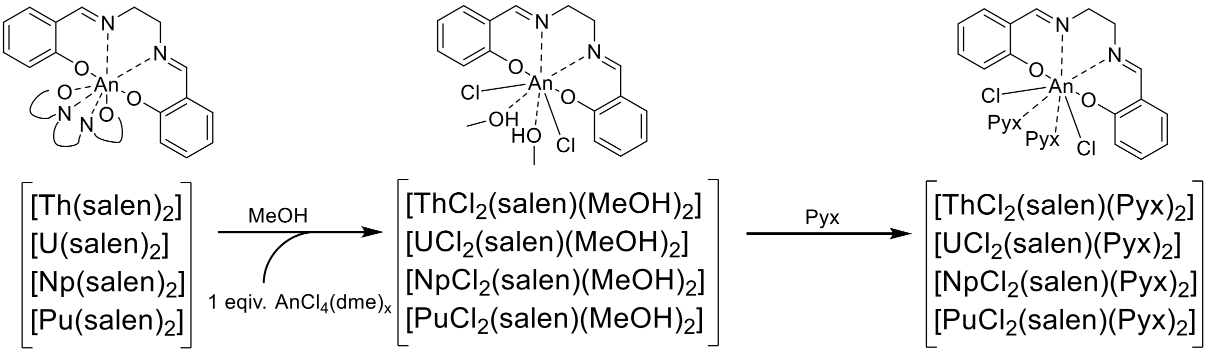

Figure S2. General synthesis of bis- and monosalen complexes of tetravalent actinides.

Synthetic descriptions, Elemental Analysis, IR bands and NMR shifts

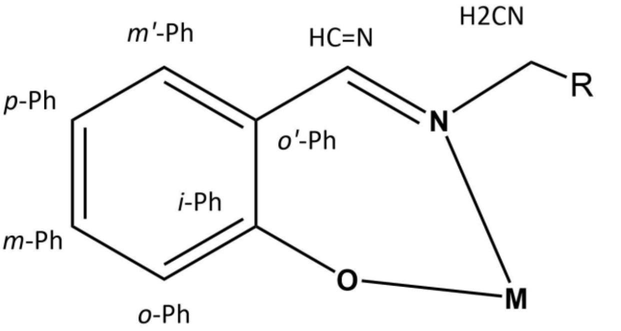

Figure S3. NMR notation scheme of the salen ligand bound in [AnCl$_2$(salen)(py)$_2$].

[An(salen)₂]

[Th(salen)$_2$]. 53.6 mg of H_2salen (0.2 mmol) and 21.6 mg of sodium methanolate (0.4 mmol) were dissolved in 4.0 mL of methanol. Adding 1.0 mL of methanol solution containing 55.6 mg [$ThCl_4$(dme)$_2$] (0.1 mmol) into the ligand solution induced an immediate fading of the solutions yellow colour and the precipitation of a white powder in several minutes. The powder was separated from the mother liquor, and washed three times with 3 mL of methanol and twice with 3 mL of *n*-pentane. Drying the washed powder *in vacuo* resulted in 74 mg of the white [Th(salen)$_2$] (yield 95%, yield calculations are always based on employed amount of metal). Elemental analysis: Anal. Calcd. for $ThN_4O_4C_{32}H_{28}$ (%): C 50.2, N 7.3, H 3.6, found; C 50.1, N 7.3, H 3.6. IR (cm^{-1}): 739 (vs), 752 (vs), 788 (s), 795 (s), 854 (s), 885 (m), 905 (s), 945 (m), 979 (w), 987 (m), 1027 (s), 1032 (s), 1042 (s), 1091 (m), 1123 (s), 1146 (s), 1196 (s), 1245 (m), 1262 (m), 1304 (vs), 1327 (s), 1339 (m), 1392 (s), 1444 (s), 1468(s), 1543 (vs), 1595 (s), 1617 (vs), 2639 (m), 2774 (m), 2850 (m), 2896 (s), 2930 (s), 2980 (m), 3022 (s), 3045 (m), 3061 (m), 3235 (w). ^{1}H NMR (400 MHz, thf-*d*$_8$) δ 8.35 (s, 4H, HC=N), 7.12 (dd, *J* = 7.6, 1.9 Hz, 4H, *m'*-PhH), 7.03 (ddd, *J* = 8.7, 7.0, 1.9 Hz, 4H, *m*-PhH), 6.41 (t, *J* = 7.3 Hz, 4H, *p*-PhH), 6.28 (d, *J* = 8.3 Hz, 4H, *o*-PhH), 4.25 (s, 8H, H2CN). ^{13}C NMR (101 MHz, thf-d$_8$) δ 166.19 (C=N), 165.49 (*i*-Ph), 134.26 (*m'*-Ph), 133.59 (*m*-Ph), 122.50 (*o'*-Ph), 119.78 (*o*-Ph), 114.72 (*p*-Ph), 62.97 (CH2N).

[U(salen)$_2$]. 107 mg of H_2salen (0.4 mmol) and 43.2 mg of sodium methanolate (0.8 mmol) were dissolved in 5.0 mL methanol. Adding 4.0 mL of methanol solution containing 72.6 mg UCl_4 (0.2 mmol) into the ligand solution induced an immediate colour change of the solution to reddish brown and the precipitation of a brown powder in several minutes. The powder was separated from the mother liquor, and washed three times with 3 mL of methanol and twice with 3 mL of n-pentane. Drying the washed powder *in vacuo* resulted in 124 mg of the brown compound [U(salen)$_2$] (yield 84%). Elemental analysis: Anal. Calcd. for $UN_4O_4C_{32}H_{28}$ (%): C 49.8, N 7.2, H 3.6, found; C 49.5, N 7.2, H 3.6. IR (cm^{-1}): 738 (vs), 751 (vs), 789 (m), 796 (m), 853 (s), 887 (m), 906 (s), 945 (m), 976 (w), 984 (m), 1027 (s), 1042 (s), 1091 (m), 1121 (m), 1146 (s), 1198 (s), 1245 (m), 1263 (m), 1296 (s), 1326 (m), 1336 (w), 1391 (s), 1438 (s), 1443 (s), 1468 (s), 1544 (s), 1594 (m), 1619 (s), 2771 (w), 2815 (m), 2849 (m), 2895 (s), 2930 (s), 2977 (w), 3012 (m), 3022 (m), 3045 (m), 3061 (m). ^{1}H NMR (600 MHz, thf-d$_8$) δ 15.65 (t, 4H, m-PhH), 14.38 (d, J = 7.1 Hz, 4H, o-PhH), 12.07 (d, J = 7.8 Hz, 4H, m'-PhH), 11.39 (s, 4H, HC=N), 10.79 (t, J = 8.7 Hz, 4H, p-PhH), -37.66 (s, 8H, H2CN). 13C NMR (151 MHz, thf) δ 183.18 (o'-Ph), 177.86 (o-Ph), 163.93 (i-Ph), 139.47 (m-Ph), 128.87 (p-Ph), 128.48 (m'-Ph), 85.42 (CH=N), -38.06 (CH2N).

[Np(salen)$_2$]. 28.2 mg of H_2salen (0.105 mmol) and 11.4 mg of sodium methanolate (0.211 mmol) were dissolved in 3 mL of methanol. Adding 2 mL of methanol solution containing 29.4 mg [$NpCl_4$(dme)$_2$] (0.052 mmol) into the ligand solution induced an immediate colour change of the solution to light beige and the precipitation of a beige powder in several minutes. The powder was separated from the mother liquor, and washed three times with 3 mL of methanol and twice times with 3 mL of n-pentane. Drying the washed powder *in vacuo* resulted in 38.1 mg of [Np(salen)$_2$] (yield 93%). IR (cm^{-1}): 739 (vs), 752 (vs), 792 (m), 797 (m), 853 (s), 889 (m), 907 (s),947 (m), 976 (w), 984 (m), 1027 (s), 1043 (s), 1092 (m), 1122 (s), 1146 (vs), 1199 (s), 1218 (vw), 1244 (m), 1264 (m), 1298 (vs), 1326 (m), 1337 (m), 1393 (s), 1438 (vs), 1444 (vs), 1468 (vs), 1546 (vs), 1594 (vs), 1620 (vs), 2633 (w), 2664 (vw), 2690 (vw), 2747 (w), 2771 (w), 2851 (m), 2896 (vs), 2931 (s), 2977 (m), 3011 (s), 3022 (s), 3046 (s),

3061 (s), 3096 (w), 3144 (w), 3189 (vw), 3238 (w). ^{1}H NMR (400 MHz, thf-d_8) δ 15.86 (d, J = 20.4 Hz, 8H, o/m-PhH), 11.29 (d, J = 5.8 Hz, 8H, p/m'-PhH), 10.90 (s, 4H, HC=N), -38.75 (s, 8H, H2CN). ^{13}C NMR (101 MHz, thf) δ 204.94 (i-Ph), 150.19 (o'-Ph), 145.55 (o/m-Ph), 128.25 (m'-Ph), 126.91 (p-Ph), 121.96 (CH=N), -62.75 (CH2N).

[Pu(salen)$_2$]. A flask was charged with a solution of 3.5 mg [$PuCl_4$(dme)$_2$] (0.006 mmol) in 1 mL methanol. Adding a solution of 3.4 mg H_2salen (0.013 mmol) and 1.4 mg sodium methanolate (0.026 mmol) in 2 mL methanol resulted in a colour change from initial yellow to orange and the precipitation of an orange powder. The powder was separated from the mother liquor, and washed three times with 3 mL of methanol and twice with 3 mL of n-pentane. Drying the washed powder under inert atmosphere resulted in 3.6 mg of [Pu(salen)$_2$] (yield 75%). IR (cm^{-1}): 739 (vs), 751 (vs), 791 (m), 798 (m), 852 (m), 864 (w), 890 (m), 907 (m), 947 (m), 975 (w), 983 (w), 1027 (m), 1031 (m), 1091 (m), 1122 (m), 1145 (vs), 1199 (s), 1217 (w), 1244 (m), 1263 (m), 1299 (vs), 1327 (m), 1337 (m), 1391 (s), 1438 (s), 1444 (s), 1467 (s), 1545 (vs), 1594 (s), 1620 (vs), 2849 (w), 2895 (m), 2912 (w), 2932 (m), 3011 (w), 3021 (m), 3045 (w), 3060 (w). ^{1}H NMR (400 MHz, thf-d_8) δ 12.09 (s, 4H, HC=N), 8.91 (ddd, J = 8.8, 7.0, 2.2 Hz, 4H, m-PhH), 8.50 (d, J = 8.2 Hz, 4H, o-PhH), 7.76 (dd, 2H, m'-PhH), 7.72 (td, J = 7.7, 1.1 Hz, 4H, p-PhH), -2.28 (s, 8H, H2CN). ^{13}C NMR (101 MHz, thf) δ 160.79 (CH=N), 158.09 (o-Ph), 137.16 (m-Ph), 130.52 (m'-Ph), 124.98 (o'-Ph), 117.48 (p-Ph), 89.70 (i-Ph), 36.18 (CH2N).

[AnCl$_2$(salen)(MeOH)$_2$]

[ThCl$_2$(salen)(MeOH)$_2$]. A flask was charged with a solution containing 30 mg [$ThCl_4$(dme)$_2$] (0.054 mmol) in 8 mL methanol. 41 mg of [Th(salen)$_2$] (0.054 mmol) was added and the suspension was stirred until the solid dissolved. The solution was evaporated by drying under a light stream of nitrogen resulting in 63 mg of a white powder [$ThCl_2$(salen)(MeOH)$_2$] (yield 92%). Clear monolithic crystals of [$ThCl_2$(salen)(MeOH)$_2$] suitable for diffraction experiments were grown by diffusion of pentane into a solution of the complex in methanol. Elemental analysis: Anal. Calcd. for $ThCl_2N_2O_4C_{18}H_{22}$ (%): C 34.1, N 4.4, H 3.5, found; C 33.4, N 4.1, H 3.3. IR (cm^{-1}): 747 (m), 768 (vs), 804 (s), 853 (m), 860 (vw), 869 (m), 899 (m), 909 (vs), 942 (wv), 956 (w), 993 (vs), 1032 (m), 1042 (m), 1074 (vw), 1095 (m), 1122 (m), 1149 (m), 1200 (m), 1253(m), 1269 (s), 1293 (vs), 1319 (w), 1344 (m) 1380 (w), 1398 (s), 1435 (m), 1446 (m), 1472 (s), 1552 (s), 1595 (s), 1617 (vs), 2741 (w), 2847 (m), 2900 (s), 2920 (s), 2947 (s), 3005 (m), 3026 (m), 3046 (m), 3105 (w), 3149 (m), 3268 (vs). ^{1}H NMR (400 MHz, CD_3OD) δ 8.54 (s, 2H, HC=N), 7.48 – 7.41 (m, 2H, p-PhH), 7.45 – 7.37 (m, 2H, m'-PhH), 6.82 (td, J = 7.4, 1.2 Hz, 2H, m-PhH), 6.81 – 6.71 (m, 2H, o-PhH), 4.13 (s, 4H, H2CN). ^{13}C NMR (101 MHz, CD_3OD) δ 168.98 (CH=N), 164.28 (o'-Ph), 136.38 (m'-Ph), 136.14 (p-Ph), 124.77 (i-Ph), 120.38 (o-Ph), 119.02 (m-Ph), 63.65 (CH2N).

The deviation in the elemental analysis (calc. vs. found) most probably stems from inorganic impurities (e.g. chloride salts), causing the lower values in the analysis.

[UCl$_2$(salen)(MeOH)$_2$]. A 2 mL solution containing 17 mg UCl_4 (0.045 mmol) in methanol was added to 34.6 mg of [U(salen)$_2$]. The suspension was stirred for several minutes while the solid dissolved and the solution changed its colour to green. The mother liquor was separated from any solid residue and was left to stand for the solvent to evaporate. This resulted in 55 mg of the green compound [UCl_2(salen)(MeOH)$_2$] (yield 96.5%). Green monolithic crystals of [UCl_2(salen)(MeOH)$_2$] suitable for diffraction experiments were grown by diffusion of pentane into a solution of the complex in methanol. Elemental analysis: Anal. Calcd. for $UCl_2N_2O_4C_{18}H_{22}$

(%): C 33.8, N 4.4, H 3.5, found; C 34.4, N 4.5, H 3.6. IR (cm^{-1}): 720 (vw), 745 (m), 769 (vs), 807 (s), 853 (m), 870 (m), 901 (m), 909 (vs), 920 (vw), 942 (vw), 957 (w), 978 (m), 1000 (vs), 1032 (m), 1043 (w), 1073 (vw), 1097 (w), 1121 (m), 1151 (m), 1155 (m), 1210 (m), 1257 (m), 1271 (s), 1282 (vs), 1319 (w), 1344 (m), 1378 (w), 1396 (s), 1433 (m), 1447 (m), 1474 (s), 1555 (s), 1595 (s), 1605 (m), 1621 (vs), 1650 (vw), 2737 (w), 2844 (m), 2904 (s), 2919 (s), 2949 (s), 2993 (w), 3028 (w), 3105 (w), 3149 (m), 3277 (vs). ^{1}H NMR (400 MHz, CD_3OD) δ 76.92, 49.07, 45.47, 44.16, 31.81, -60.00.

The deviation in the elemental analysis most probably stems from organic impurities like methanol due to insufficient drying of the [UCl_2(salen)(MeOH)$_2$] complex powder.

[$NpCl_2$(salen)(MeOH)$_2$]. A solution containing 29.4 mg of [$NpCl_4$(dme)$_2$] in 2 mL of methanol was added to a flask charged with 38 mg of compound [Np(salen)$_2$]. The suspension was stirred for several minutes while the solid was dissolved and a colour change of the solution to greenish beige occured. An addition of about 15 mL pentane induced precipitation of a beige powder. Drying the powder under inert atmosphere resulted in 54 mg of the beige complex [$NpCl_2$(salen)(MeOH)$_2$] (yield 67%). Greenish beige monolithic crystals of [$NpCl_2$(salen)(MeOH)$_2$] suitable for diffraction experiments were grown by diffusion of pentane into a solution of the complex in methanol. IR (cm^{-1}): 670 (vw), 768 (vs), 806 (s), 854 (m), 870 (m), 909 (vs), 942 (w), 958 (m), 978 (m), 1000 (vs), 1032 (m), 1044 (m), 1073 (vw), 1097 (m), 1122 (m), 1151 (m), 1155 (m), 1208 (m), 1220 (w), 1254 (m), 1270 (s), 1285 (vs), 1320 (w), 1344 (m), 1375 (w), 1397 (s), 1434 (m), 1439 (vw), 1446 (m), 1473 (s), 1554 (s), 1594 (s), 1620 (vs), 2732 (w), 2844 (m), 2903 (s), 2921 (s), 2950 (s), 2998 (m), 3028 (w), 3045 (w), 3280 (vs). ^{1}H NMR (400 MHz, CD_3OD) δ 71.87, 52.14, 44.12, 39.60, 30.55, -53.00.

Unfortunately, an assignment of the proton signals was not possible for both, [UCl_2(salen)(MeOH)$_2$] and [$NpCl_2$(salen)(MeOH)$_2$], as the paramagnetism of U(IV) and Pu(IV) caused massive signal broadening here. In addition, no ^{13}C resonances were accessible for those complexes, most probably due to paramagnetic relaxation enhancement (PRE).

[$PuCl_2$(salen)(MeOH)$_2$]. A solution containing 7.6 mg of [$PuCl_4$(dme)$_2$] (0.013 mmol) in 2 mL of methanol was added to a flask charged with 10.6 mg of compound [Pu(salen)$_2$] (0.013 mmol). The suspension was stirred for several minutes while the solid was dissolved and the solution changed its colour to red. Evaporating the solvent under inert atmosphere resulted in 14.3 mg of the red compound [$PuCl_2$(salen)(MeOH)$_2$] (yield 83%). Red monolithic crystals of [$PuCl_2$(salen)(MeOH)$_2$] suitable for diffraction experiments were grown by diffusion of pentane into a solution of the complex in methanol. IR (cm^{-1}): 673 (vw), 758 (vs), 768 (vs), 807 (s), 854 (m), 869 (m), 910 (vs), 942 (w), 960 (m), 978 (m), 995 (vs), 1030 (m), 1044 (m), 1074 (w), 1097 (m), 1103 (w), 1124 (m), 1149 (m), 1155 (m), 1209 (m), 1220 (w), 1252 (m), 1271 (s), 1288 (vs), 1321 (w), 1345 (m), 1371 (w), 1398 (s), 1436 (m), 1472 (s), 1552 (s), 1594 (s), 1618 (vs), 2844 (m), 2904 (s), 2922 (s), 2950 (s), 2997 (m), 3026 (m), 3047 (m), 3069 (m), 3103 (m), 3312 (vs). ^{1}H NMR (400 MHz, CD_3OD) δ 24.96, 14.24, 13.30, 11.22, 11.20, 10.32, 10.31, 10.29, 4.87, -1.68. ^{13}C NMR (400 MHz, CD_3OD) δ 149.60, 133.22, 130.13, 26.59.

In a subsequent step, $[AnCl_2(salen)(MeOH)_2]$ can be converted into $[AnCl_2(salen)(Pyx)_2]$.

Table S1: Synthesis details and yields for bulk powders of An(IV)[a] complexes $[AnCl_2(salen)(Pyx)_2]$ **1**, **2**, **5**, **6**, **9** and **10**.

	starting compound $[AnCl_2(salen)(MeOH)_2]$	solvent	yield
$[ThCl_2(salen)(py)_2]$ **1**	46.4 mg (0.07 mmol)	5.5 mL py	53.3 (81.1%)
$[UCl_2(salen)(py)_2]$ **2**	65.1 mg (0.10 mmol)	6.0 mL py	61.4 (82.2%)
$[ThCl_2(salen)(4\text{-}pic)_2]$ **5**	41.2 mg (0.06 mmol)	3.5 mL 4-pic	49.1 (86.9%)
$[UCl_2(salen)(4\text{-}pic)_2]$ **6**	71.2 mg (0.11 mmol)	3.5 mL 4-pic	84.8 (89.2%)
$[ThCl_2(salen)(3,5\text{-}lut)_2]$ **9**	22.0 mg (0.03 mmol)	13.0 mL 3,5-lut	27.2 (99.2%)
$[UCl_2(salen)(3,5\text{-}lut)_2]$ **10**	17.7 mg (0.04mmol)	13.0 mL 3,5-lut	21.8 (94.7%)

a) No yields were calculated for the complexes containing transuranic elements, since sub-miligram amounts of starting compounds were employed for the synthesis of a few single crystals.

*$[AnCl_2(salen)(py)_2]$ (**1-4**)*.

$[ThCl_2(salen)(py)_2]$ (***1***). Elemental analysis: Anal. Calcd. for $ThN_4O_2Cl_2C_{26}H_{24}$ (%): C 42.9, N 7.7, H 3.3, found; C 43.1, N 7.9, H 3.5. IR (cm^{-1}): 654 (vw), 697 (vs), 705 (s), 740 (m), 748 (s), 756 (vs), 766 (s), 803 (m), 862 (m), 893 (w), 910 (m), 958 (m), 991 (m), 1005 (m), 1036 (s), 1049 (m), 1071 (m), 1102 (m), 1125 (m), 1152 (m), 1208 (m), 1216 (m), 1234 (w), 1252 (m), 1270 (m), 1289 (vs), 1306 (w), 1324 (vw), 1344 (m), 1403 (m), 1418 (m),1437 (s), 1473 (s), 1553 (m), 1572 (w), 1581 (m), 1597 (s), 1623 (s), 2851 (m), 2918 (s), 3064 (m). ^{1}H NMR (400 MHz, Pyridine-d_5) δ 8.53 (s, 2H, HC=N), 7.55 – 7.51 (m, 2H, *p*-PhH), 7.51 – 7.48 (m, 2H, *o*-PhH), 7.09 (dd, *J* = 7.9, 1.1 Hz, 2H, *m'*-PhH), 6.92 (td, *J* = 7.4, 1.2 Hz, 2H, *m*-PhH), 4.49 (s, 4H, H2CN). ^{13}C NMR (101 MHz, Pyridine-d_5) δ 166.15 (CH=N), 163.84 (*i*-Ph), 135.36 (*p*-Ph), 124.75 (*o'*-Ph), 119.52 (*m'*-Ph), 118.45 (*m*-Ph), 63.11 (CH2N).

$[UCl_2(salen)(py)_2]$ (***2***). Elemental analysis: Anal. Calcd. for $UN_4O_2Cl_2C_{26}H_{24}$ (%): C 42.6, N 7.6, H 3.3, found; C 42.5, N 7.8, H 3.4. IR (cm^{-1}): 654 (vw), 697 (vs), 704 (s), 739 (m), 748 (s), 756 (vs), 766 (s), 771 (s), 806 (s), 865 (m), 911 (s), 960 (m), 991 (m), 1004 (m), 1036 (s), 1051 (m), 1071 (m), 1104 (m), 1124 (m), 1153 (m), 1216 (m), 1234 (w), 1252 (m), 1278 (vs), 1344 (m),1400 (m), 1414 (m),1437 (s), 1475 (s), 1557 (m), 1572 (w), 1581 (m), 1597 (s), 1626 (s), 2853 (m), 2913 (s), 3063 (m). ^{1}H NMR (400 MHz, Pyridine-d_5) δ 75.67 (s, 2H, *o*-PhH), 49.52 (s, 2H, CH=N), 44.75 (d, 2H, *m*-PhH), 43.83 (d, 2H, *m'*-PhH), 31.51 (d, 2H, *p*-PhH), -59.00 (s, 4H, H2CN). ^{13}C NMR (101 MHz, Pyridine-d_5) δ 192.48 (*m*-Ph), 186.07 (*m'*-Ph), 173.43 (*p*-Ph).

Not all ^{13}C resonances were accessible, most probably due to paramagnetic relaxation enhancement (PRE).

$[NpCl_2(salen)(py)_2]$ (***3***). A. IR (cm^{-1}): 654 (vw), 697 (vs), 703 (s), 746 (vs), 756 (vs), 772 (s), 800 (s), 910 (s), 947 (w), 958 (m), 991 (m), 1005 (m), 1036 (s), 1050 (m), 1071 (m), 1101 (m), 1125 (m), 1149 (s), 1180 (m), 1212 (m), 1252 (m), 1282 (vs), 1326 (w), 1340 (m), 1400 (m), 1409 (m), 1440 (s), 1474 (s), 1556 (m), 1595 (s), 1626 (vs), 2853 (m), 2921 (s), 2952 (w), 3021 (w), 3062 (w).

[$PuCl_2$(salen)(py)$_2$] (***4***). A. IR (cm^{-1}): 681 (vw), 704 (vs), 745 (s), 760 (vs), 806 (vs), 863 (m), 912 (vs), 958 (m), 991 (m), 1006 (m), 1036 (s), 1050 (m), 1071 (m), 1099 (m), 1126 (m), 1150 (s), 1168 (m), 1210 (m), 1252 (m), 1290 (vs), 1343 (m), 1382 (m), 1401 (m), 1440 (s), 1474 (s), 1555 (s), 1595 (s), 1626 (vs), 2853 (m), 2921 (m), 2923 (s), 3019 (w), 3048 (w).

[$AnCl_2$(salen)(pic)$_2$] (***5***-***8***).

[$ThCl_2$(salen)(4-pic)$_2$] (***5***). Elemental analysis: Anal. Calcd. for $ThN_4O_2Cl_2C_{28}H_{28}$ (%): C 44.5, N 7.4, H 3.7, found; C 44.1, N 7.6, H 3.9. IR (cm^{-1}):671 (vw), 681 (vw), 722 (m), 742 (s), 157 (vs), 802 (vs), 854 (m), 908 (s), 951 (m), 981 (w), 996 (m), 1014 (s), 1036 (m), 1045 (m), 1071 (w), 1098 (w), 1125 (m), 1151 (m), 1201 (m), 1216 (w), 1221 (w), 1230 (w), 1253 (w), 1292 (vs), 1307 (m), 1343 (m), 1351 (vw), 1378 (m), 1404 (m), 1417 (m), 1436 (m), 1448 (m), 1474 (s), 1504 (w), 1554 (m), 1596 (s), 1603 (m), 1618 (vs). 2852 (m), 2921 (s), 2952 (m), 3024 (w), 3062 (w).

[UCl_2(salen)(4-pic)$_2$] (***6***). Elemental analysis: Anal. Calcd. for $UN_4O_2Cl_2C_{28}H_{28}$ (%): C 44.2, N 7.4, H 3.7, found; C 45.1, N 7.8, H 4.0. IR (cm^{-1}): 679 (w), 705 (w), 711 (w), 725 (w), 742 (s), 757 (vs), 803 (vs), 856 (m), 909 (vs), 953 (m), 993 (m), 1013 (m), 1034 (m),1047 (m), 1071 (w), 1099 (m), 1125 (m), 1150 (m), 1169 (w), 1175 (w), 1208 (m), 1221 (m), 1228 (m), 1256 (m), 1282 (vs), 1301 (m), 1330 (w), 1342 (m), 1351 (w), 1401 (m), 1413 (m), 1446 (m), 1473 (m), 1502 (w), 1548 (m), 1559 (m), 1595 (s), 1624 (vs), 2854 (m), 2916 (s), 3024 (w), 3063(w).

The deviations in the elemental analysis of [$ThCl_2$(salen)(4-pic)$_2$] and [UCl_2(salen)(4-pic)$_2$] reflect the challenges for an exact drying of the bulk powders. In [$ThCl_2$(salen)(4-pic)$_2$] the elemental analysis points to a slightly too intense drying, i.e. a loss of about 0.1 eq. 4-pic per complex molecule. On the other hand, a residue of remaining 0.25 eq. 4-pic per [UCl_2(salen)(4-pic)$_2$] are most probably the reason for the slightly higher values in elemental analysis of [UCl_2(salen)(4-pic)$_2$].

[$NpCl_2$(salen)(4-pic)$_2$] (***7***). A. IR (cm^{-1}): 704 (w), 722 (m), 742 (m), 756 (vs), 803 (vs), 855 (m), 909 (s), 940 (w), 955 (m), 980 (w), 992 (m), 1013 (s), 1036 (m), 1049 (m), 1071 (w), 1099 (m), 1125 (m), 1151 (m), 1208 (m), 1228 (m), 1256 (m), 1283 (vs), 1302 (m), 1343 (m), 1352 (w), 1379 (w), 1403 (m) 1415 (s), 1475 (m), 1502 (w), 1558 (m), 1594 (s), 1603 (m), 1617 (vs), 1626 (vs), 2852 (m), 2921 (s), 2951 (w), 3063 (vw).

[$PuCl_2$(salen)(4-pic)$_2$] (***8***). A. IR (cm^{-1}): 711 (w), 723 (m), 742 (m), 757 (vs), 8045 (vs), 855 (m), 862 (m), 911 (s), 937 (w), 956 (m), 991 (m), 1014 (s), 1036 (m), 1048 (m), 1071 (w), 1099 (m), 1126 (m), 1152 (m), 1208 (m), 1226 (m), 1255 (m), 1289 (vs), 1343 (m), 1352 (w), 1380 (w), 1402 (m) 1414 (s), 1448 (m), 1475 (m), 1501 (w), 1556 (m), 1596 (s), 1627 (vs), 2854 (m), 2920 (s), 3064 (vw).

[AnCl$_2$(salen)(3,5-lut)$_2$] (***9-12***).

[ThCl$_2$(salen)(3,5-lut)$_2$] (**9**). Elemental analysis: Anal. Calcd. for $ThN_4O_2Cl_2C_{30}H_{32}$ (%): C 46.0, N 7.2, H 4.1, found; C 48.6, N 8.4, H 5.2. IR (cm^{-1}): 703 (s), 708 (s), 745 (s), 748 (s), 775 (s), 799 (m), 856 (m), 864 (m), 870 (w), 890 (m), 909 (s), 929 (w), 940 (w), 954 (m), 982 (w), 995 (m), 1035 (m), 1047 (m), 1076 (w), 1099 (m), 1126 (m), 1147 (s), 1173 (m), 1202 (m), 1252 (m), 1268 (m), 1292 (vs), 1307 (m), 1341 (m), 1347 (w), 1379 (m), 1402 (m), 1411 (m), 1439 (s), 1444 (s), 1473 (s), 1555 (m), 1622 (vs), 1651 (w), 2853 (s), 2922 (vs), 2955 (m).

[UCl$_2$(salen)(3,5-lut)$_2$] (**10**). Elemental analysis: Anal. Calcd. for $UN_4O_2Cl_2C_{30}H_{32}$ (%): C 45.6, N 7.1, H 4.1, found; C 49.5, N 8.2, H 5.4. IR (cm^{-1}): 703 (s), 708 (s), 743 (s), 745 (s), 760 (vs), 775 (s), 801 (m), 804 (m), 858 (m), 870 (w), 896 (m), 910 (s), 940 (w), 956 (m), 980 (w), 992 (m), 1037 (m), 1048 (m), 1078 (w), 1100 (m), 1126 (m), 1148 (s), 1166 (w), 1178 (m), 1205 (m), 1211 (m), 1252 (m), 1257 (m), 1282 (vs), 1340 (m), 1347 (w), 1379 (m), 1400 (m), 1408 (m), 1441 (s), 1476 (s), 1559 (m), 1595 (s) 1622 (vs), 1651 (w), 2870 (s), 2915 (vs), 2956 (m).

The strong deviations in [ThCl$_2$(salen)(3,5-lut)$_2$] and [UCl$_2$(salen)(3,5-lut)$_2$] are due to incomplete drying of the bulk powders, as 3,5-lutidin is on the one hand embedded in the crystal packing with half a molecule per complex molecule, and has on the other hand a high boiling point of 172°C. This causes increased values for C, N, and H.

[NpCl$_2$(salen)(3,5-lut)$_2$] (**11**). A. IR (cm^{-1}): 704 (s), 708 (s), 743 (s), 746 (s), 760 (vs), 774 (s), 800 (m), 860 (m), 871 (w), 910 (s), 957 (m), 980 (w), 992 (m), 1037 (m), 1049 (m), 1078 (vw), 1100 (m), 1126 (m), 1148 (s), 1167 (w), 1177 (m), 1204 (m), 1211 (m), 1252 (m), 1256 (m), 1283 (vs), 1300 (m), 1340 (m), 1347 (w), 1380 (m), 1400 (m), 1409 (m), 1442 (s), 1475 (s), 1558 (m), 1595 (s), 1626 (vs), 1653 (w), 2869 (s), 2918 (vs), 2957 (m).

[PuCl$_2$(salen)(3,5-lut)$_2$] (**12**). A. IR (cm^{-1}): 710 (vs), 743 (s), 760 (s), 774 (s), 802 (m), 857 (m), 911 (s), 958 (m), 980 (w), 992 (m), 1034 (s), 1049 (m), 1078 (w), 1100 (m), 1126 (m), 1148 (s), 1168 (m), 1204 (w), 1211 (m), 1235 (m), 1252 (m), 1257 (m), 1287 (vs), 1321 (w), 1340 (m), 1380 (m), 1400 (m), 1410 (m), 1423 (m), 1443 (s), 1475 (s), 1557 (m), 1580 (m), 1595 (s) 1626 (vs), 1679 (w), 2860 (s), 2920 (vs), 2957 (m), 3018 (m).

Crystal structure

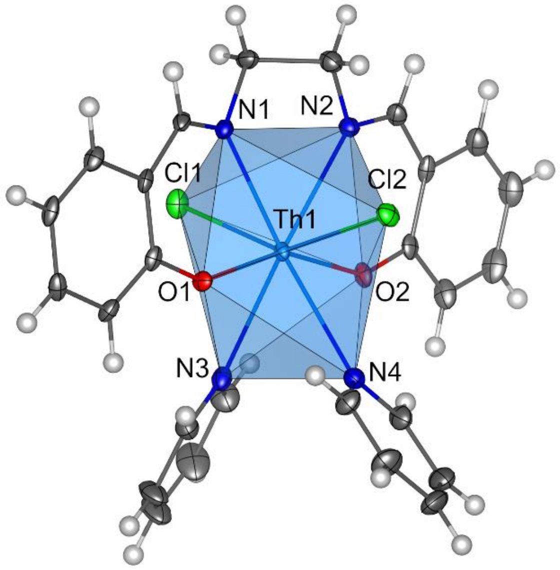

Figure S3. Molecular structure of [$ThCl_2$(salen)(py)$_2$] (**1**). Thermal anisotropic displacement displayed at 50% probability level. (Color code: carbon dark grey, nitrogen blue, oxygen red, thorium and coordination polyhedron light blue.)

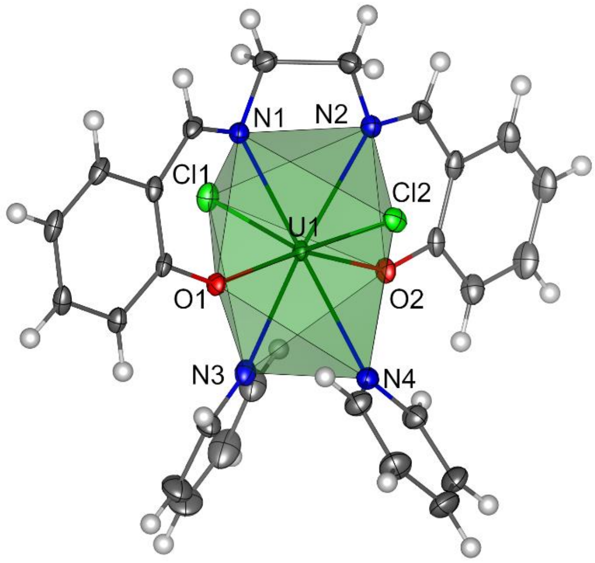

Figure S4. Molecular structure of [UCl_2(salen)(py)$_2$] (**2**). Thermal anisotropic displacement displayed at 50% probability level. (Color code: carbon dark grey, nitrogen blue, oxygen red, uranium and coordination polyhedron dark green.)

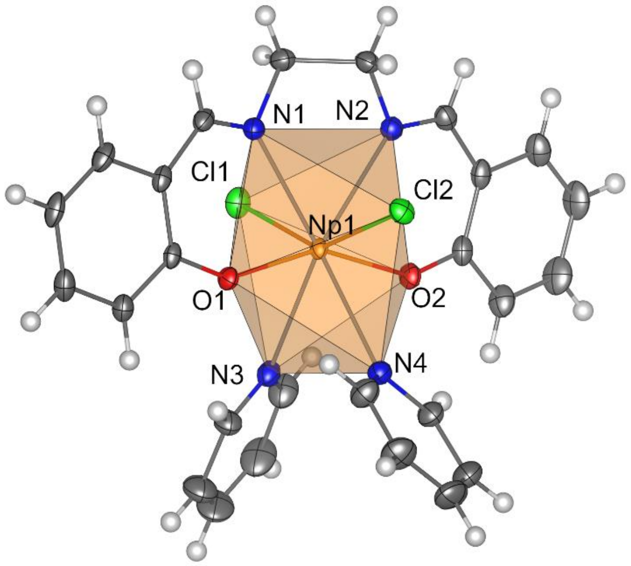

Figure S5. Molecular structure of [$NpCl_2$(salen)$(py)_2$] (**3**). Thermal anisotropic displacement displayed at 50% probability level. (Color code: carbon dark grey, nitrogen blue, oxygen red, neptunium and coordination polyhedron orange.)

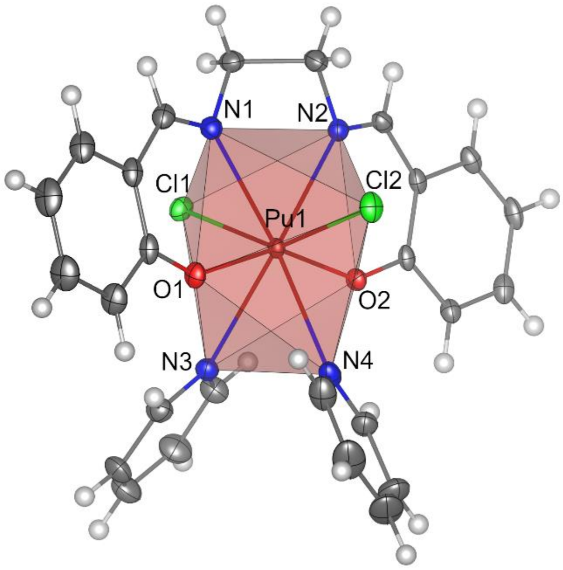

Figure S6. Molecular structure of [$PuCl_2$(salen)$(py)_2$] (**4**). Thermal anisotropic displacement displayed at 50% probability level. (Color code: carbon dark grey, nitrogen blue, oxygen red, plutonium and coordination polyhedron dark red.)

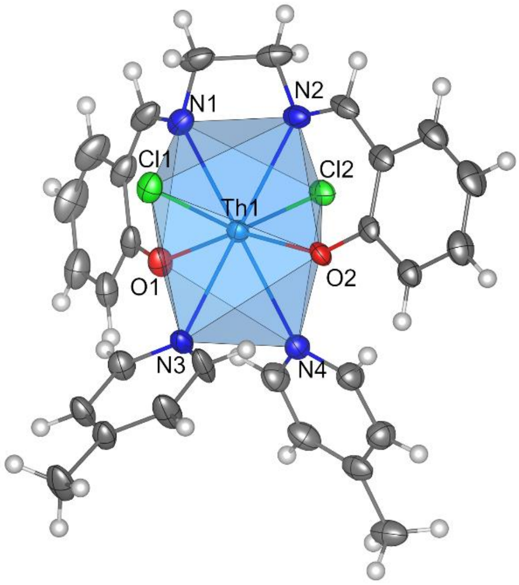

Figure S7. Molecular structure of $[ThCl_2(salen)(4\text{-pic})_2]$ (**5**). Thermal anisotropic displacement displayed at 50% probability level. (Color code: carbon dark grey, nitrogen blue, oxygen red, thorium and coordination polyhedron light blue.)

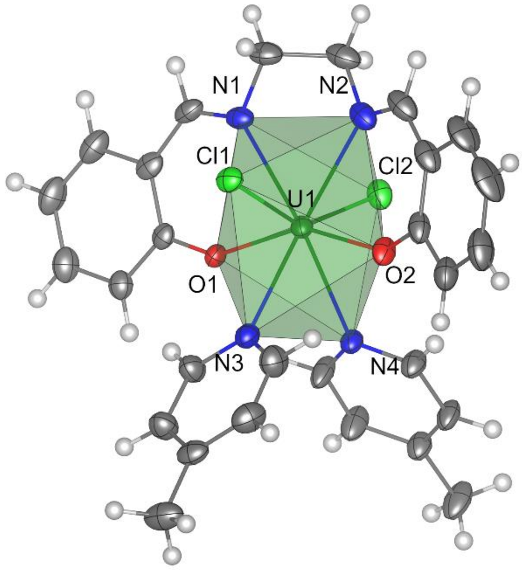

Figure S8. Molecular structure of $[UCl_2(salen)(4\text{-pic})_2]$ (**6**). Thermal anisotropic displacement displayed at 50% probability level. (Color code: carbon dark grey, nitrogen blue, oxygen red, uranium and coordination polyhedron dark green.)

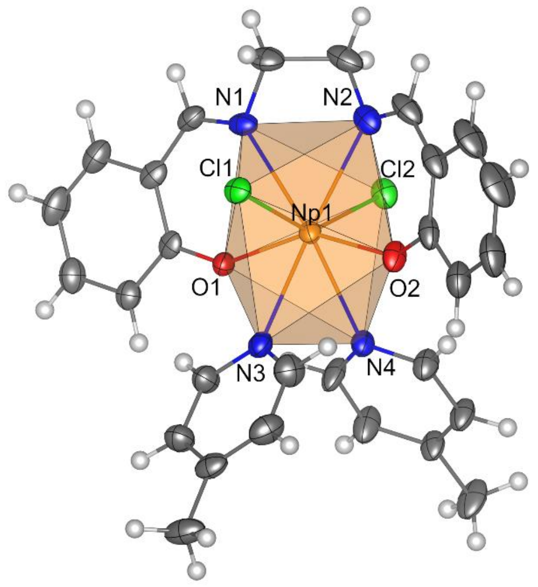

Figure S9. Molecular structure of [$NpCl_2$(salen)(4-pic)$_2$] (**7**). Thermal anisotropic displacement displayed at 50% probability level. (Color code: carbon dark grey, nitrogen blue, oxygen red, neptunium and coordination polyhedron orange.)

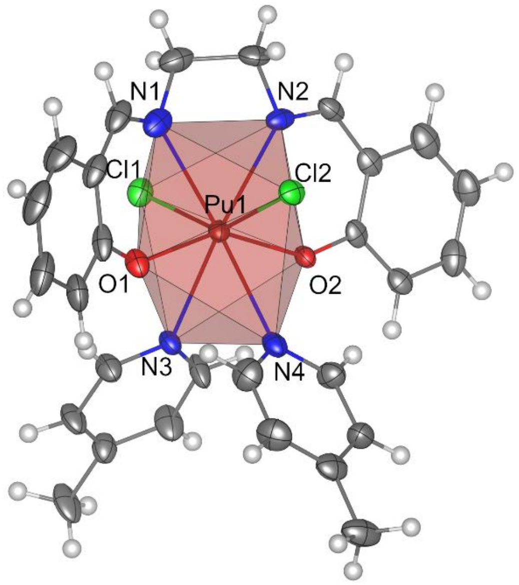

Figure S10. Molecular structure of [$PuCl_2$(salen)(4-pic)$_2$] (**8**). Thermal anisotropic displacement displayed at 50% probability level. (Color code: carbon dark grey, nitrogen blue, oxygen red, plutonium and coordination polyhedron dark red.)

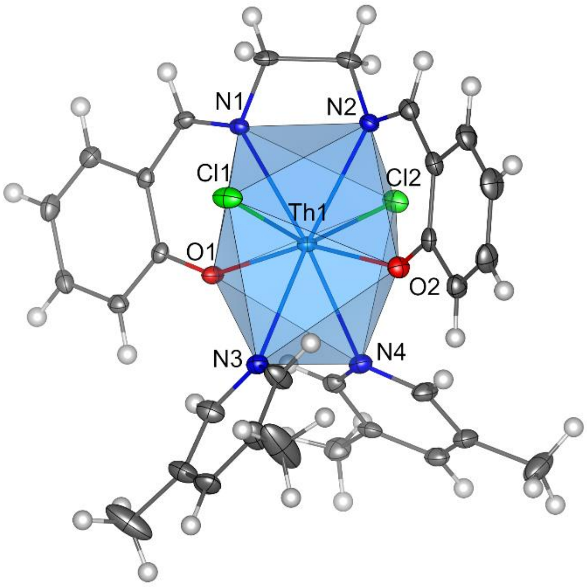

Figure S11. Molecular structure of [$ThCl_2$(salen)(3,5-lut)$_2$] (**9**). Thermal anisotropic displacement displayed at 50% probability level. (Color code: carbon dark grey, nitrogen blue, oxygen red, thorium and coordination polyhedron light blue.)

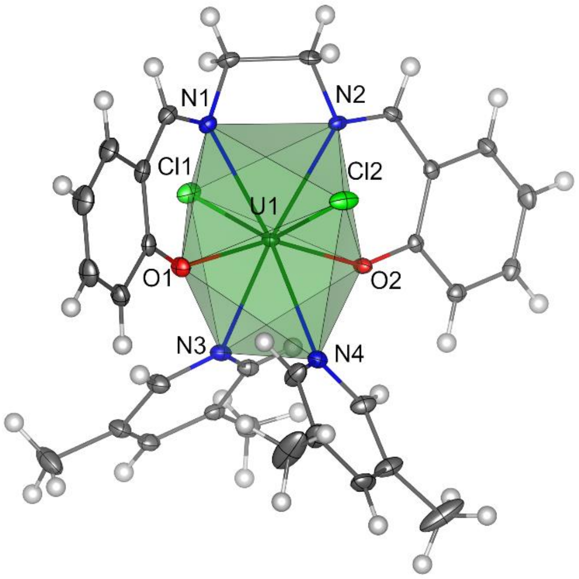

Figure S12. Molecular structure of [UCl_2(salen)(3,5-lut)$_2$] (**10**). Thermal anisotropic displacement displayed at 50% probability level. (Color code: carbon dark grey, nitrogen blue, oxygen red, uranium and coordination polyhedron dark green.)

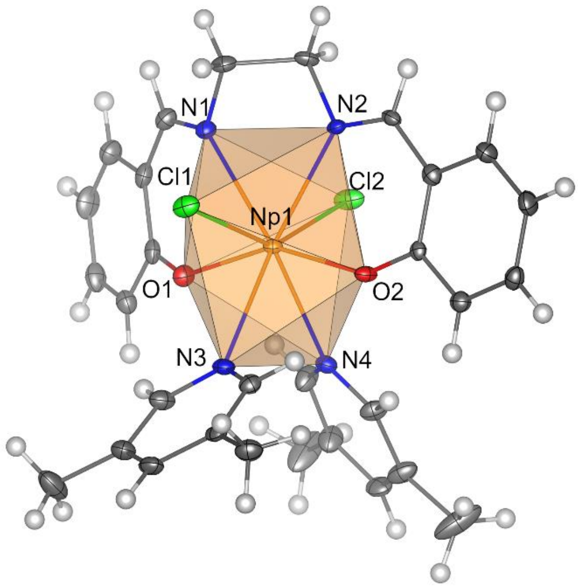

Figure S13. Molecular structure of [NpCl$_2$(salen)(3,5-lut)$_2$] (**10**). Thermal anisotropic displacement displayed at 50% probability level. (Color code: carbon dark grey, nitrogen blue, oxygen red, neptunium and coordination polyhedron orange.)

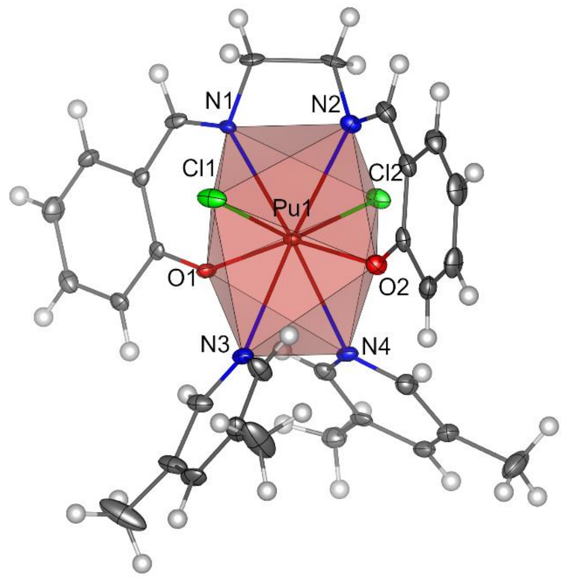

Figure S14. Molecular structure of [PuCl$_2$(salen)(3,5-lut)$_2$] (**12**). Thermal anisotropic displacement displayed at 50% probability level. (Color code: carbon dark grey, nitrogen blue, oxygen red, plutonium and coordination polyhedron dark red.)

Structure parameters derived from SC-XRD and QC calculations

Hydrogen bonds and π interactions

Table S2. Hydrogen bond parameters in [UCl_2(salen)(py)$_2$] derived from SC-XRD.

**Hydrogen bond D–H···A**	**d(H···A) in Å**	**d(D···A) in Å**	**Angle D–H···A in °**
C9–H9B···Cl2	2.85	3.40	115.7
C22–H22···O1	2.54	3.10	118.6
C21–H21···O2	2.49	3.07	120.1

Table S3. Hydrogen bond parameters in [UCl_2(salen)(pic)$_2$] derived from SC-XRD.

**Hydrogen bond D–H···A**	**d(H···A) in Å**	**d(D···A) in Å**	**Angle D–H···A in °**
C9–H9B···Cl2	2.85	3.45	118.2
C22–H22···O1	2.57	3.19	122.7
C23–H23···O1	2.40	3.03	123.3
C17–H17···O2	2.42	3.04	123.3
C28–H28···O2	2.59	3.19	121.5

Table S4. Hydrogen bond parameters in [UCl_2(salen)(lut)$_2$] derived from SC-XRD.

**Hydrogen bond D–H···A**	**d(H···A) in Å**	**d(D···A) in Å**	**Angle D–H···A in °**
C8–H8B···Cl2	2.76	3.36	119.5
C21–H21···O1	2.57	3.12	117.1
C24–H24···O1	2.58	3.19	122.1
C17–H17···O2	2.58	3.15	118.7
C28–H28···O2	2.51	3.14	123.2

Table S5. π···π interaction parameters Pyx···Pyx in [UCl_2(salen)(Pyx)$_2$] derived from SC-XRD.

**Complex**	**d(Cg1···Cg2) in Å**	**Angle (plane1-plane2) in °**
[UCl_2(salen)(py)$_2$] (**2**)	4.56	36.6
[UCl_2(salen)(pic)$_2$] (**6**)	4.02	5.7

Coordinating bond lengths from SC-XRD and QC calculations

Table S6. Coordinating bond lengths in $[UCl_2(salen)(Pyx)_2]$ derived from SC-XRD structures (black values) as well as from DFT structure optimisations (grey values).

Bond U–X X=…	Bond length in Å in exp. and opt. $[UCl_2(salen)(Pyx)_2]$, with Pyx=					
	**py (2)**	py opt (**2a**)	**pic (6)**	pic opt (**6a**)	**lut (10)**	lut opt (**10a**)
Cl1	2.701	2.674	2.718	2.676	2.698	2.673
Cl2	2.725	2.676	2.719	2.679	2.725	2.674
Cl3	2.701					
Cl4	2.738					
O1	2.158	2.181	2.168	2.182	2.173	2.181
O2	2.185	2.183	2.184	2.185	2.186	2.185
O3	2.168					
O4	2.171					
N1, salen	2.586	2.562	2.562	2.564	2.579	2.563
N2, salen	2.594	2.568	2.579	2.573	2.593	2.564
N3, salen	2.570					
N4, salen	2.590					
N1, pyx	2.636	2.649	2.639	2.638	2.646	2.656
N2, pyx	2.683	2.649	2.669	2.639	2.650	2.658
N3, pyx	2.652					
N4, pyx	2.654					

Table S7. Measured angles in $[UCl_2(salen)(Pyx)_2]$ derived from SC-XRD structures as well as from DFT structure optimisations.

Angle	Angle in ° in $[UCl_2(salen)(Pyx)_2]$, with Pyx= …		
	**py (2)**	**pic (6)**	**lut (10)**
Cl1-U-Cl2	147.82	146.7	145.4
	148.35		
$N1_{salen}$-U-$N2_{salen}$	66.66	69.53	70.14
	66.83		
$O1_{salen}$-U-$O2_{salen}$	154.61	152.25	153.61
	155.09		
N_{Pyx1}-U-N_{Pyx2}	66.46	67.12	66.27
	65.97		
$Phenyl_{salen}$-$Phenyl_{salen}$	174.55	156.92	158.00
	179.60		
Pyx-Pyx	66.00	80.42	83.00
	69.35		
Calc. Pyx-Pyx	24.69 (in **10a**)	55.15 (in **6a**)	53.36 (in **2a**)

Table S8. Coordinating bond lengths in $[AnCl_2(salen)(Pyx)_2]$ (An = Th – Pu) derived from SC-XRD structures (black values) as well as from DFT structure optimisations (grey values).

**Bond An–X, X=…**	**Bond length in Å in exp. and opt. $[AnCl_2(salen)(pic)_2]$, with An=**							
	Th,exp (**5**)	Th,calc (**5a**)	Pa, calc	U,exp (**6**)	U,calc (**6a**)	Np,exp (**7**)	Np,calc (**7a**)	Pu,exp (**8**)	Pu,calc (**8a**)
Cl1	2.760	2.733	2.694	2.718	2.676	2.698	2.676	2.672	2.643
Cl2	2.764	2.735	2.694	2.719	2.679	2.704	2.678	2.679	2.672
O1	2.228	2.252	2.210	2.168	2.182	2.161	2.175	2.155	2.159
O2	2.258	2.253	2.213	2.184	2.185	2.186	2.175	2.178	2.175
N1, salen	2.599	2.621	2.588	2.562	2.564	2.542	2.558	2.535	2.546
N2, salen	2.622	2.623	2.590	2.579	2.573	2.568	2.559	2.538	2.563
N1, Pyx	2.667	2.686	2.663	2.639	2.638	2.618	2.607	2.603	2.604
N2, Pyx	2.707	2.691	2.663	2.669	2.639	2.646	2.611	2.639	2.632

Results from quantum chemical calculations

Geometry optimisations in comparison with SC-XRD results

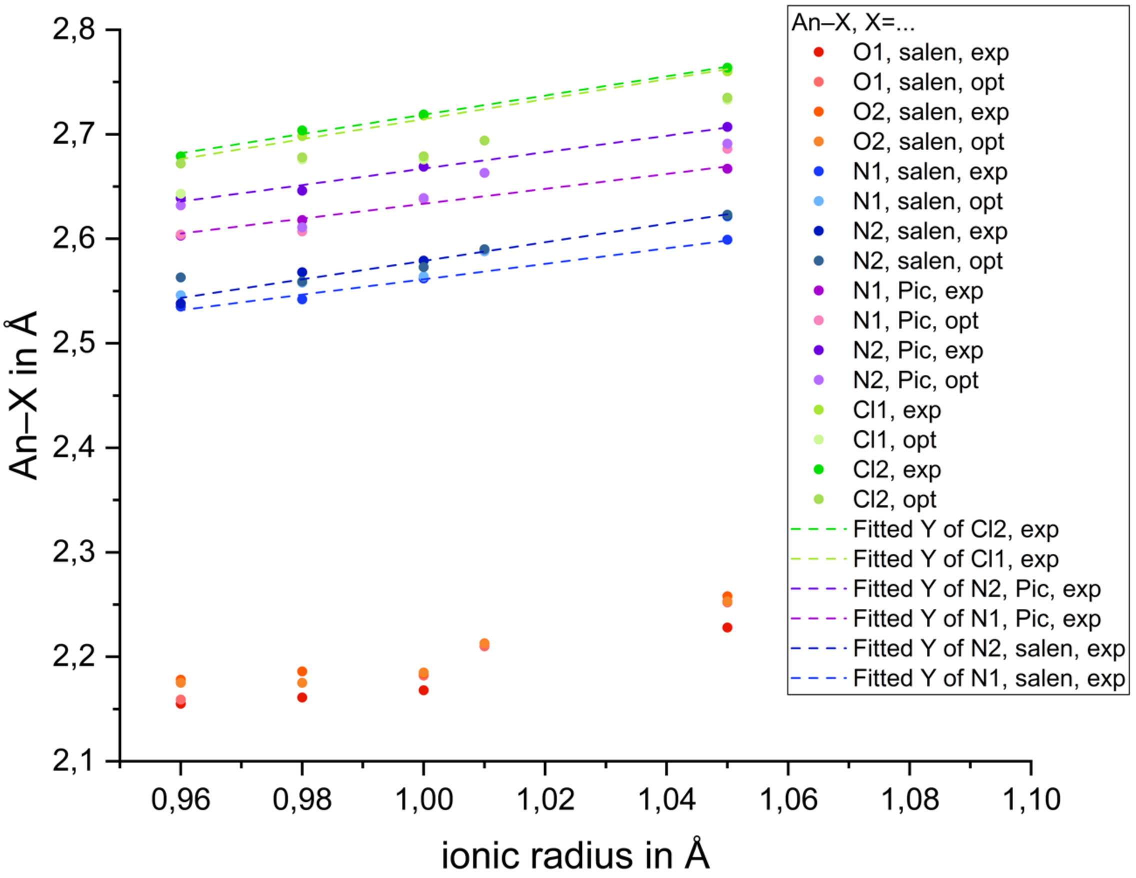

Figure S15. Plot of coordinating bond lengths in [$AnCl_2$(salen)(pic)$_2$], determined from SC-XRD as well as structure optimisations, vs. the Shannon ionic radii for CN 8 and the respective linear fits (dashed lines) for An–Cl and An–N.

NBO analysis

Table S9. Calculated charges of uranium and coordinating atoms in [UCl_2(salen)(Pyx)$_2$].

**Atom**	**Atom charge in [UCl_2(salen)(Pyx)$_2$], with Pyx= …**		
	**py** (**2a**)	**pic** (**6a**)	**lut** (**10a**)
**U**	1.215	1.219	1.205
Cl1	-0.414	-0.416	-0.411
Cl2	-0.415	-0.418	-0.412
**ø Cl**	-0.414	-0.417	-0.412
O1	-0.721	-0.720	-0.721
O2	-0.720	-0.721	-0.720
**ø O**	-0.721	-0.720	-0.721
N1, salen	-0.537	-0.537	-0.537
N2, salen	-0.537	-0.536	-0.538
**ø N_{salen}**	-0.537	-0.536	-0.537
N1, pyx	-0.523	-0.531	-0.514
N2, pyx	-0.522	-0.531	-0.515
**ø N_{pyx}**	-0.523	-0.531	-0.514

Table S10. Calculated charges of the actinide and coordinating atoms in [$AnCl_2$(salen)(pic)$_2$].

**Atom**	**Atom charge in [$AnCl_2$(salen)(pic)$_2$] with An=…**				
	**Th** (**5**)	**Pa**	**U** (**6**)	**Np** (**7**)	**Pu** (**8**)
**An**	**1.310**	**1.239**	**1.219**	**1.262**	**1.329**
Cl1	-0.381	-0.382	-0.416	-0.452	-0.481
Cl2	-0.383	-0.382	-0.418	-0.449	-0.457
**ø Cl**	**-0.382**	**-0.382**	**-0.417**	**-0.450**	**-0.469**
O1	-0.794	-0.753	-0.720	-0.722	-0.721
O2	-0.794	-0.752	-0.721	-0.720	-0.717
**ø O**	**-0.794**	**-0.753**	**-0.720**	**-0.721**	**-0.719**
N1, salen	-0.567	-0.551	-0.537	-0.533	-0.534
N2, salen	-0.568	-0.551	-0.536	-0.535	-0.537
**ø N_{salen}**	**-0.567**	**-0.536**	**-0.536**	**-0.534**	**-0.535**
N1, pyx	-0.555	-0.536	-0.531	-0.538	-0.535
N2, pyx	-0.556	-0.536	-0.531	-0.540	-0.554
**ø N_{pyx}**	**-0.555**	**-0.536**	**-0.531**	**-0.539**	**-0.544**

Delocalisation indices (DI's)

Table S11. Calculated delocalization indices (DI's) for coordinating bonds in $[UCl_2(salen)(Pyx)_2]$.

**Bond U–X X=…**	**DI in $[UCl_2(salen)(Pyx)_2]$, with Pyx= …**		
	**py** (**2a**)	**pic** (**6a**)	**lut** (**10a**)
Cl1	0.57	0.56	0.57
Cl2	0.57	0.57	0.57
O1	0.61	0.61	0.61
O2	0.61	0.61	0.61
N1, salen	0.31	0.30	0.31
N2, salen	0.31	0.31	0.31
N1, pyx	0.26	0.26	0.26
N2, pyx	0.26	0.27	0.26

Table S12. Calculated delocalization indices (DI's) for coordinating bonds in $[AnCl_2(salen)(pic)_2]$.

**Bond An–X X=…**	**DI in $[AnCl_2(salen)(pic)_2]$ with An=…**				
	**Th** (**5**)	**Pa**	**U** (**6**)	**Np** (**7**)	**Pu** (**8**)
Cl1	0.51	0.56	0.56	0.55	0.56
Cl2	0.51	0.57	0.57	0.55	0.59
O1	0.52	0.58	0.61	0.62	0.61
O2	0.52	0.58	0.61	0.62	0.64
N1, salen	0.27	0.31	0.30	0.31	0.30
N2, salen	0.28	0.31	0.31	0.31	0.32
N1, pyx	0.24	0.25	0.26	0.27	0.25
N2, pyx	0.24	0.25	0.27	0.28	0.27

Table S13. Calculated delocalization indices (DI's) for coordinating bonds in $[ThCl_2(salen)(pic)_2]$ in three different conformations. "**1a**" represents the calculated structure of complex **1**, "single twisted" stands for changed positions of one chlorine and one 4-picoline ligand and in the "double twisted" structure, the position of both chlorine and 4-picoline ligands are exchanged.

**Bond An–X X=…**	**DI in $[ThCl_2(salen)(pic)_2]$**		
	**1a**	**Single twisted**	**Double twisted**
Cl1	0.51	0.50	0.53
Cl2	0.51	0.51	0.53
O1	0.52	0.52	0.53
O2	0.52	0.53	0.53
N1, salen	0.27	0.25	0.23
N2, salen	0.28	0.26	0.23
N1, pyx	0.24	0.26	0.25
N2, pyx	0.24	0.26	0.25

Table S14 Averaged QTAIM metrics for the An-Ligand bonds in the investigated systems. Rho is the electron density at the bond critical point (BCP). H is the energy density at the BCP and DI the delocalization index.

	Pyridin			Picolin			Lutidin		
	DI	Rho	H	DI	Rho	H	DI	Rho	H
Th-O				0.5244	0.0898	-0.0197			
Th-N_sal				0.2753	0.0501	-0.0061			
Th-N_pyx				0.2423	0.0436	-0.0041			
Th-Cl				0.5090	0.0573	-0.0107			
Pa-O				0.5798	0.0973	-0.0229			
Pa-N_sal				0.3120	0.0521	-0.0063			
Pa-N_pyx				0.2477	0.0466	-0.0049			
Pa-Cl				0.5647	0.0608	-0.0119			
U-O	0.6119	0.1028	-0.0255	0.6093	0.1025	-0.0251	0.6095	0.1025	-0.0252
U-N_sal	0.3092	0.0532	-0.0063	0.3054	0.0529	-0.0062	0.3094	0.0529	-0.0062
U-N_pyx	0.2620	0.0440	-0.0037	0.2660	0.0451	-0.0040	0.2570	0.0427	-0.0035
U-Cl	0.5666	0.0622	-0.0121	0.5634	0.0618	-0.0119	0.5711	0.0626	-0.0122
Np-O				0.6204	0.1033	-0.0250			
Np-N_sal				0.3102	0.0526	-0.0058			
Np-N_pyx				0.2720	0.0477	-0.0045			
Np-Cl				0.5529	0.0611	-0.0113			
Pu-O				0.6250	0.1031	-0.0239			
Pu-N_sal				0.3087	0.0522	-0.0054			
Pu-N_pyx				0.2601	0.0461	-0.0038			
Pu-Cl				0.5750	0.0622	-0.0114			

As the DI in itself might be an ambiguous metric for the determination of bond strengths, we have looked at the relative changes in all three presented metrics over the actinide series for all An-ligand bonds. The Figure S21 shows the four diagrams.

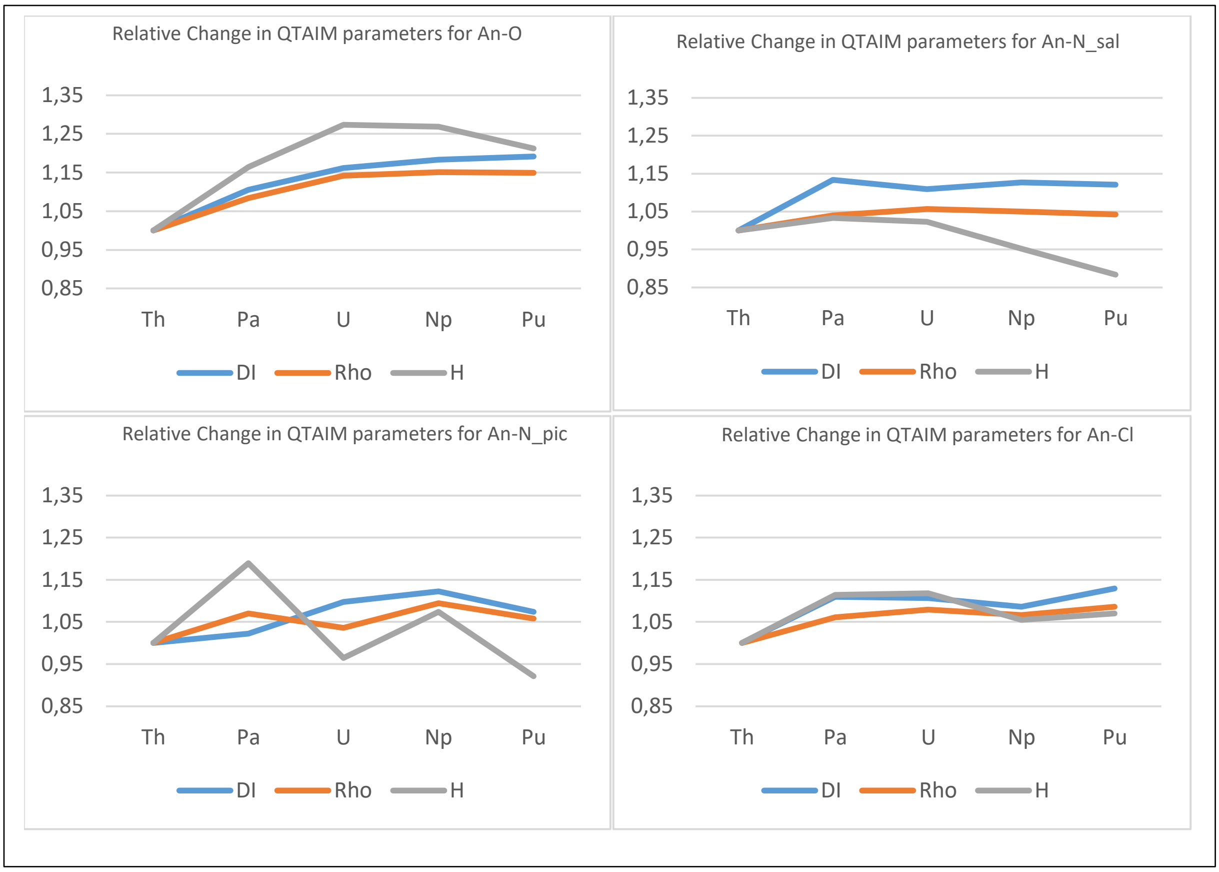

Figure S17 Relative change in QTAIM metrics for the An series of the picoline complexes.

Structure optimization after conformational changes of $[ThCl_2(salen)(pic)_2]$

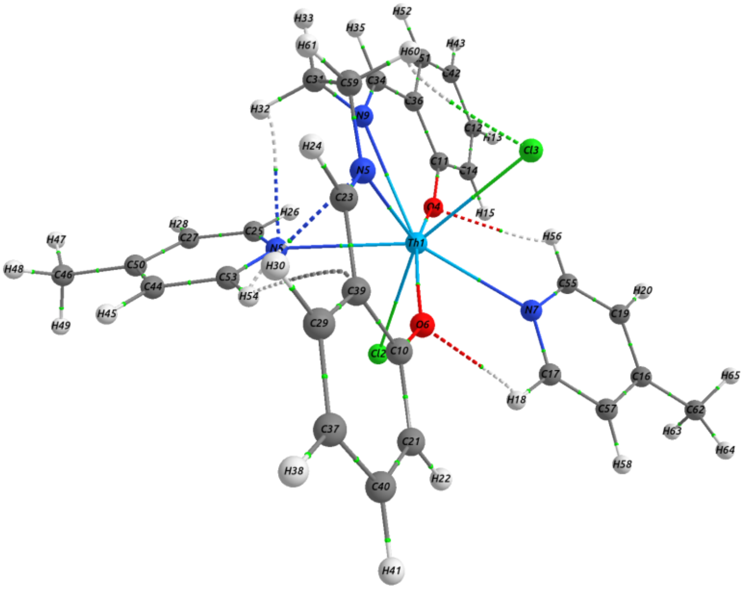

Figure S18. Optimized structure of $[ThCl_2(salen)(pic)_2]$ with changed positions of one chlorine and one 4-picoline ligand.

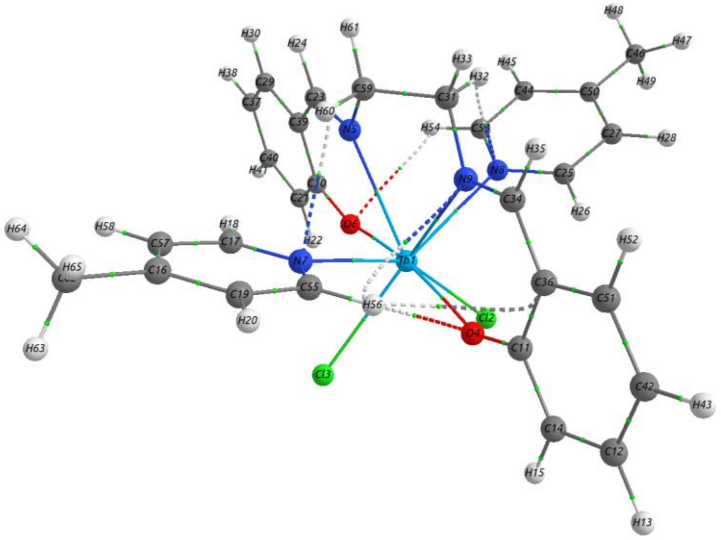

Figure S19. Optimized structure of $[ThCl_2(salen)(pic)_2]$ with changed positions of both chlorine and one 4-picoline ligands.

IR data

A series of IR spectra of the intermediate products [An(salen)$_2$] or [AnCl$_2$(salen)(MeOH)$_2$] as well as the IR spectra of [AnCl$_2$(salen)(Pyx)$_2$] (An = Th, U, Np, Pu) confirmed the isostructurality of all synthesized complexes in the solid state:

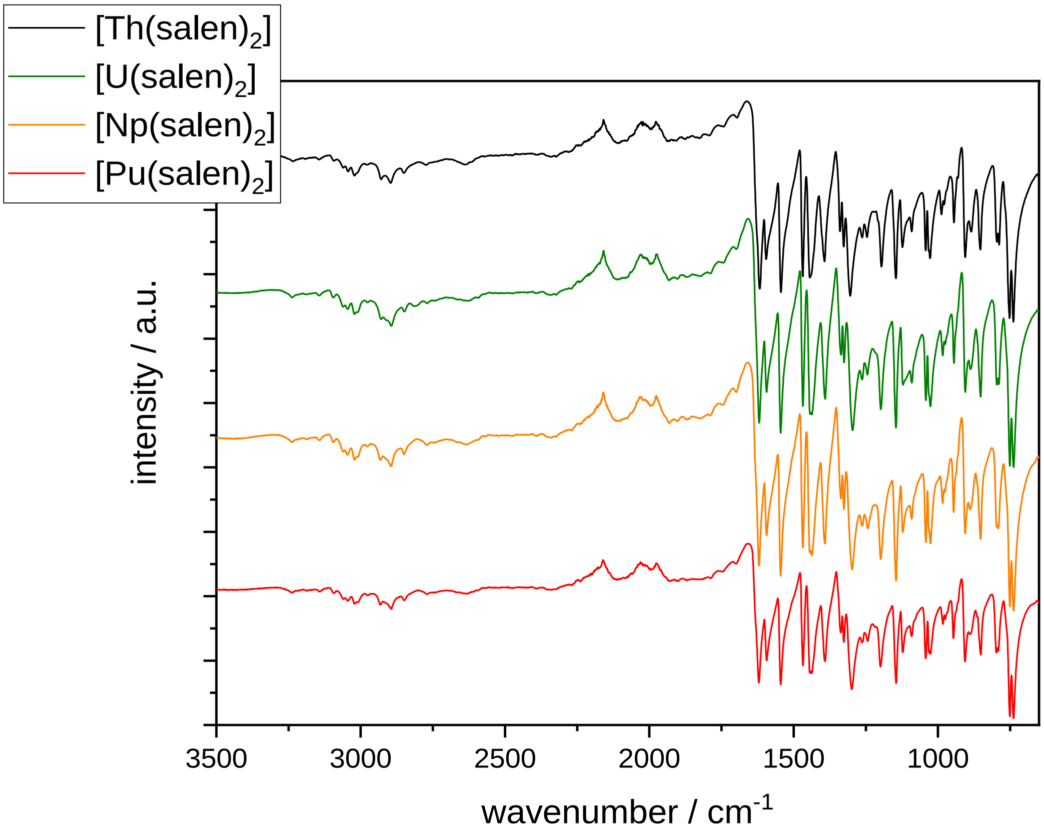

Figure S20. IR spectra of complexes [An(salen)$_2$].

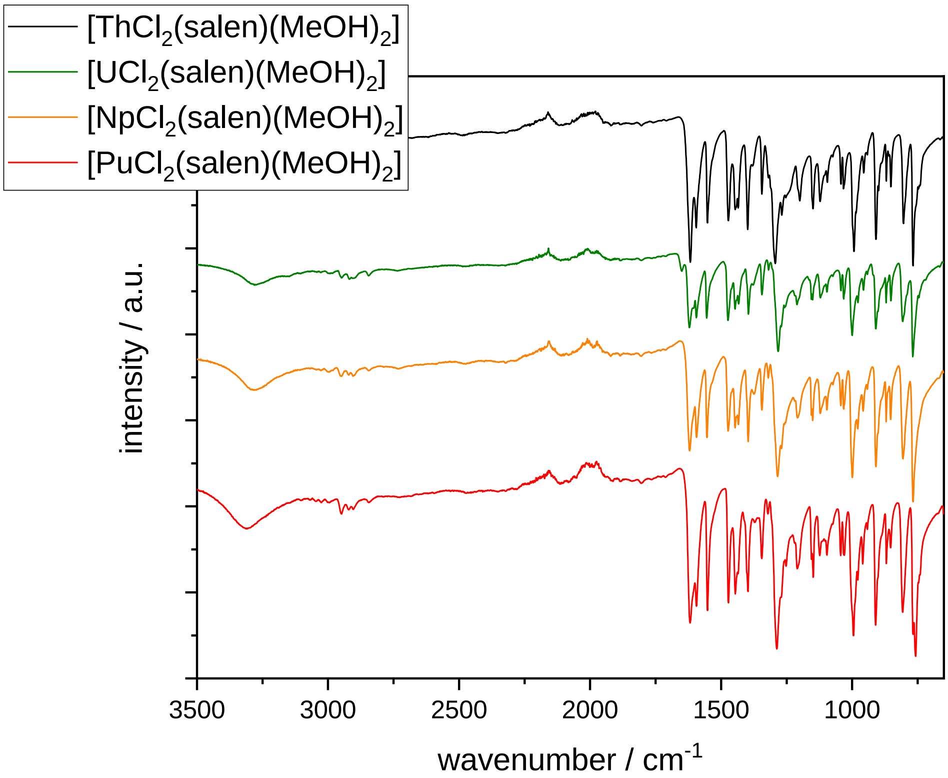

Figure S21. IR spectra of complexes [AnCl2(salen)(MeOH)2].

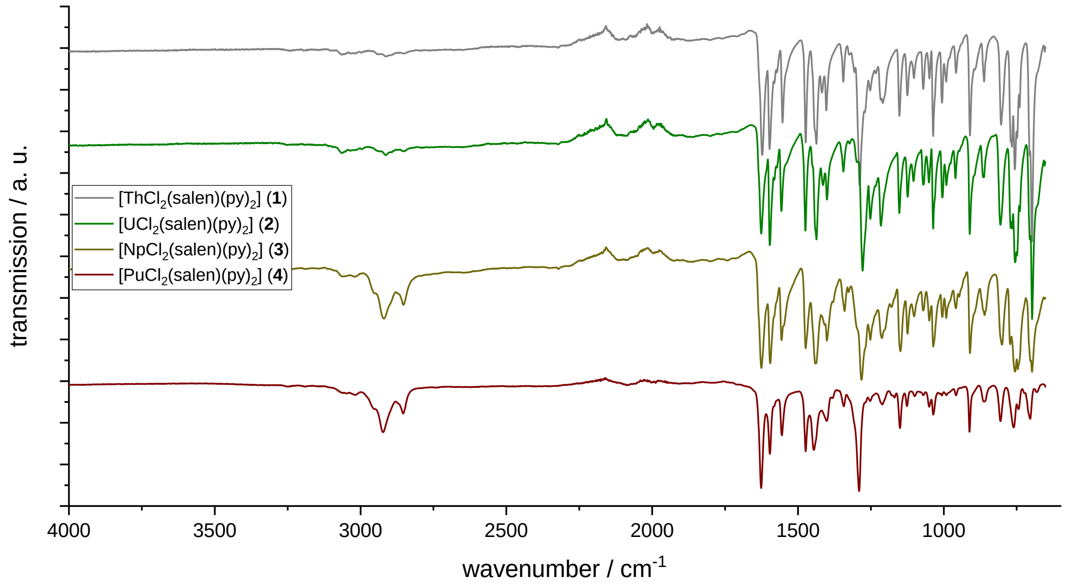

Figure S22. Solid state IR spectra of [AnCl$_2$(salen)(py)$_2$] (**1**-**4**).

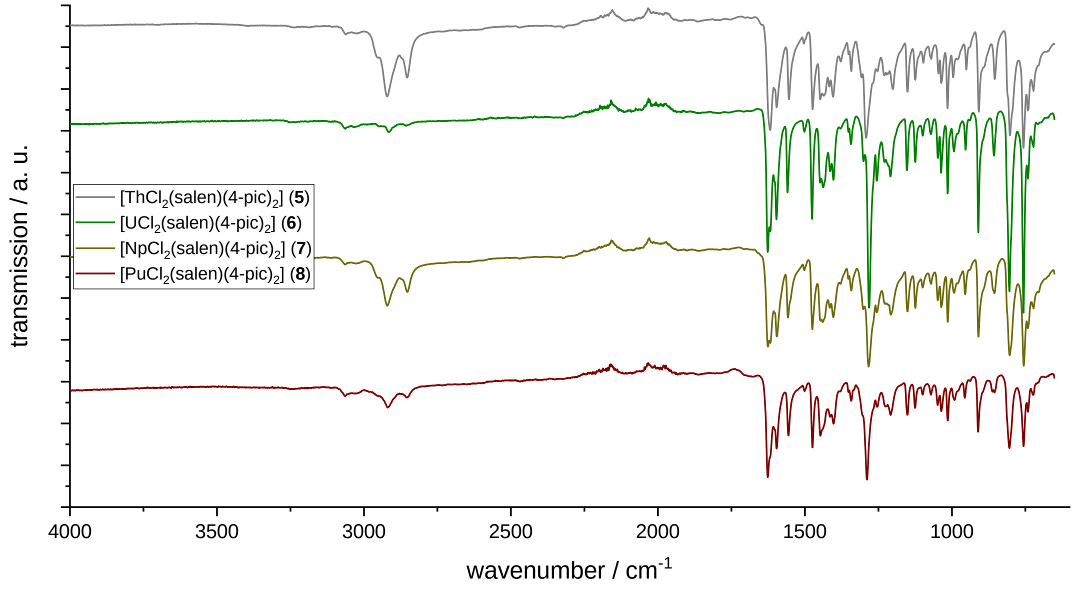

Figure S23. Solid state IR spectra of [AnCl$_2$(salen)(pic)$_2$] (**5**-**8**).

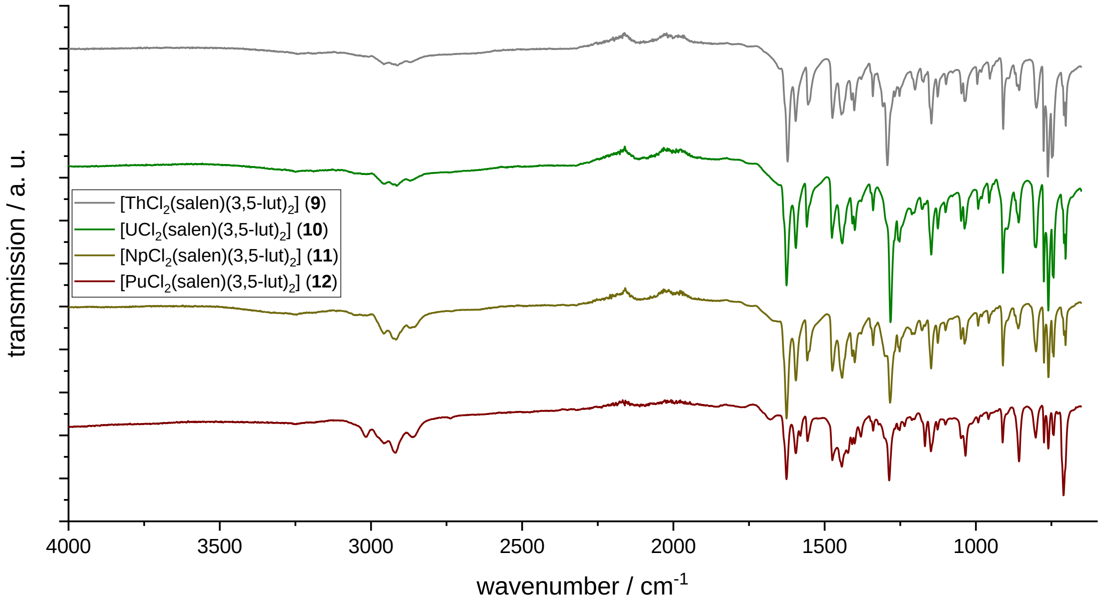

Figure S24. Solid state IR spectra of [$AnCl_2$(salen)(lut)$_2$] (**9**-**12**).

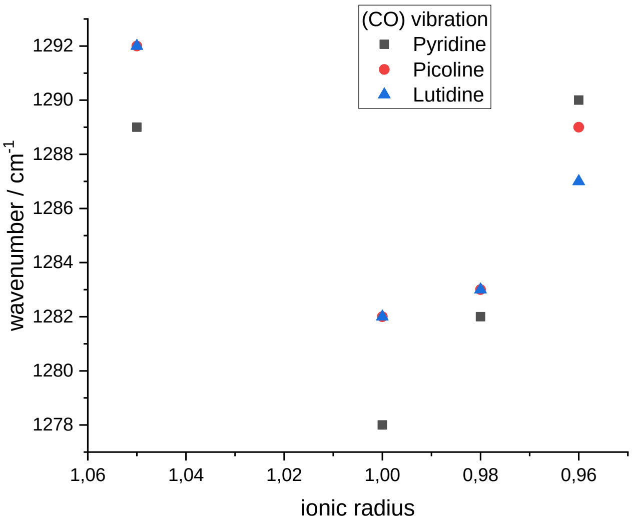

Figure S25. IR wavelengths of the carbonyl vibration of the salen ligand in [$AnCl_2$(salen)(Pyx)$_2$] (An = Th, U, Np, Pu) plotted against the ionic radius of An(IV) with CN 8. The data of the py and pic series overlap for Th, U and Np.

Table S15. IR bands of coordinating functional groups in [$AnCl_2$(salen)(Pyx)$_2$] (An = Th, U, Np, Pu).

	[$AnCl_2$(salen)(py)$_2$]		[$AnCl_2$(salen)(pic)$_2$]		[$AnCl_2$(salen)(lut)$_2$]	
Vibration	$\nu(CO)_{str.}$	$\nu(CN)_{str.}$	$\nu(CO)_{str.}$	$\nu(CN)_{str.}$	$\nu(CO)_{str.}$	$\nu(CN)_{str.}$
Th	1289	1623	1292	1618	1292	1622
U	1278	1626	1282	1624	1282	1622
Np	1282	1626	1283	1626	1283	1626
Pu	1290	1626	1289	1627	1287	1626

Powder XDR patterns of bulk powders.

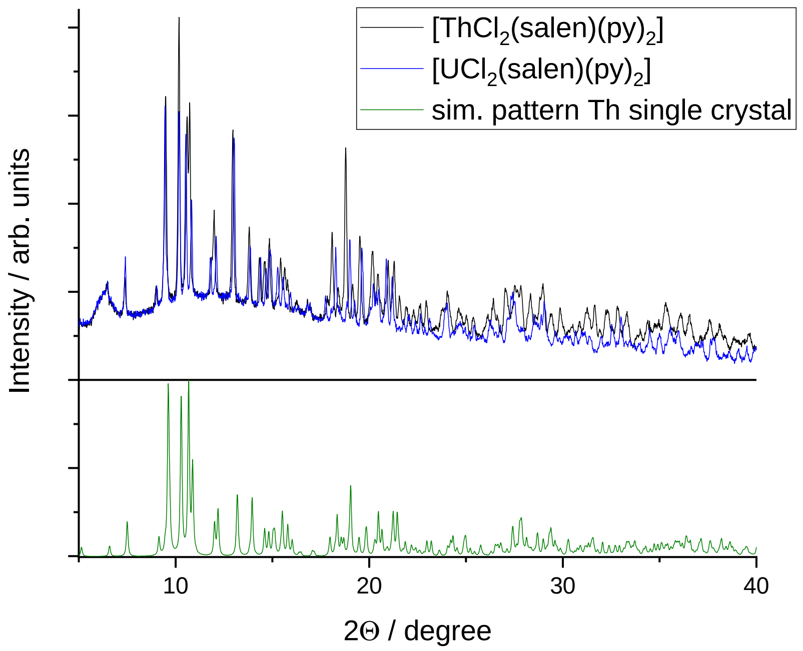

Figure S26. PXRD patterns of [AnCl$_2$(salen)(py)$_2$] (An = Th, U) (top) and the simulated pattern for the respective Th(IV) single crystal (bottom)

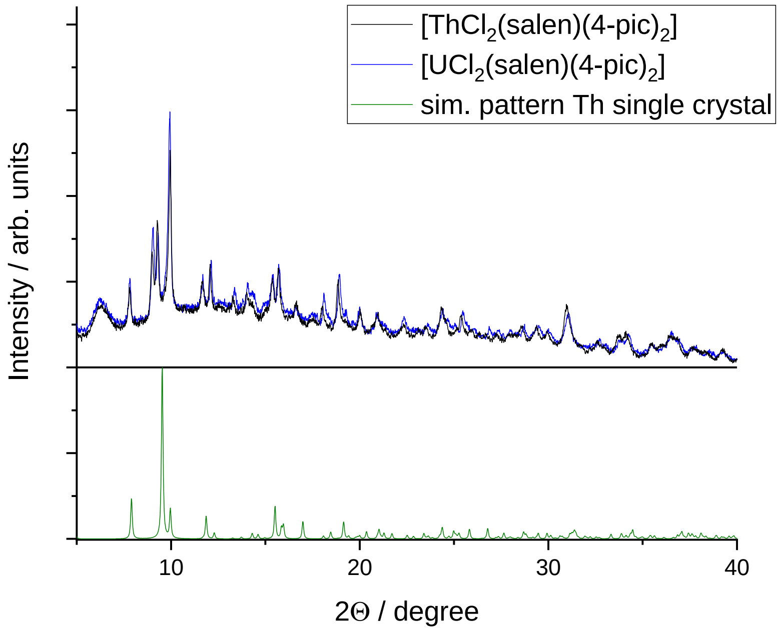

Figure S27. PXRD patterns of [AnCl$_2$(salen)(4-pic)$_2$] (An = Th, U) (top) and the simulated pattern for the respective Th(IV) single crystal (bottom)

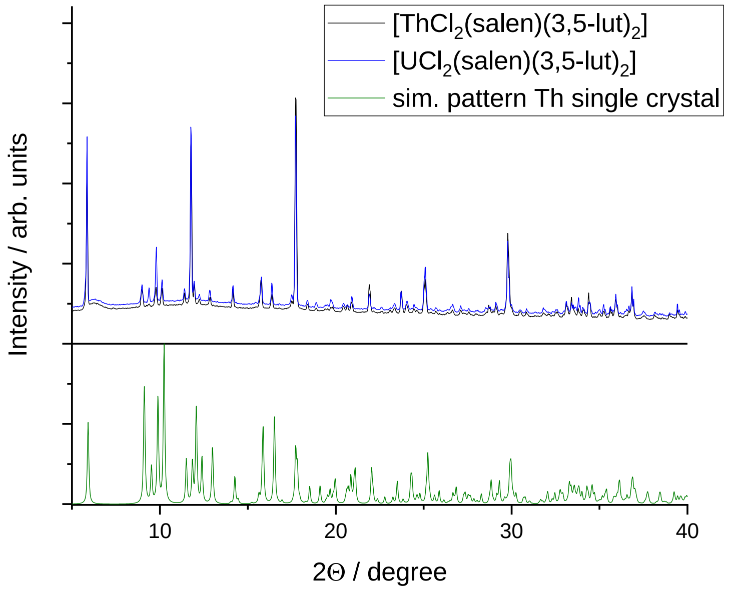

Figure S28. PXRD patterns of $[AnCl_2(salen)(3,5\text{-lut})_2]$ (An = Th, U) (top) and the simulated pattern for the respective Th(IV) single crystal (bottom).

NMR spectra

[An(salen)$_2$]

NMR spectra of the stoichiometric 2:1 (salen to An(IV)) synthesis attempts showed one set of ligand signals, confirming the 2:1 complex formation in solution.

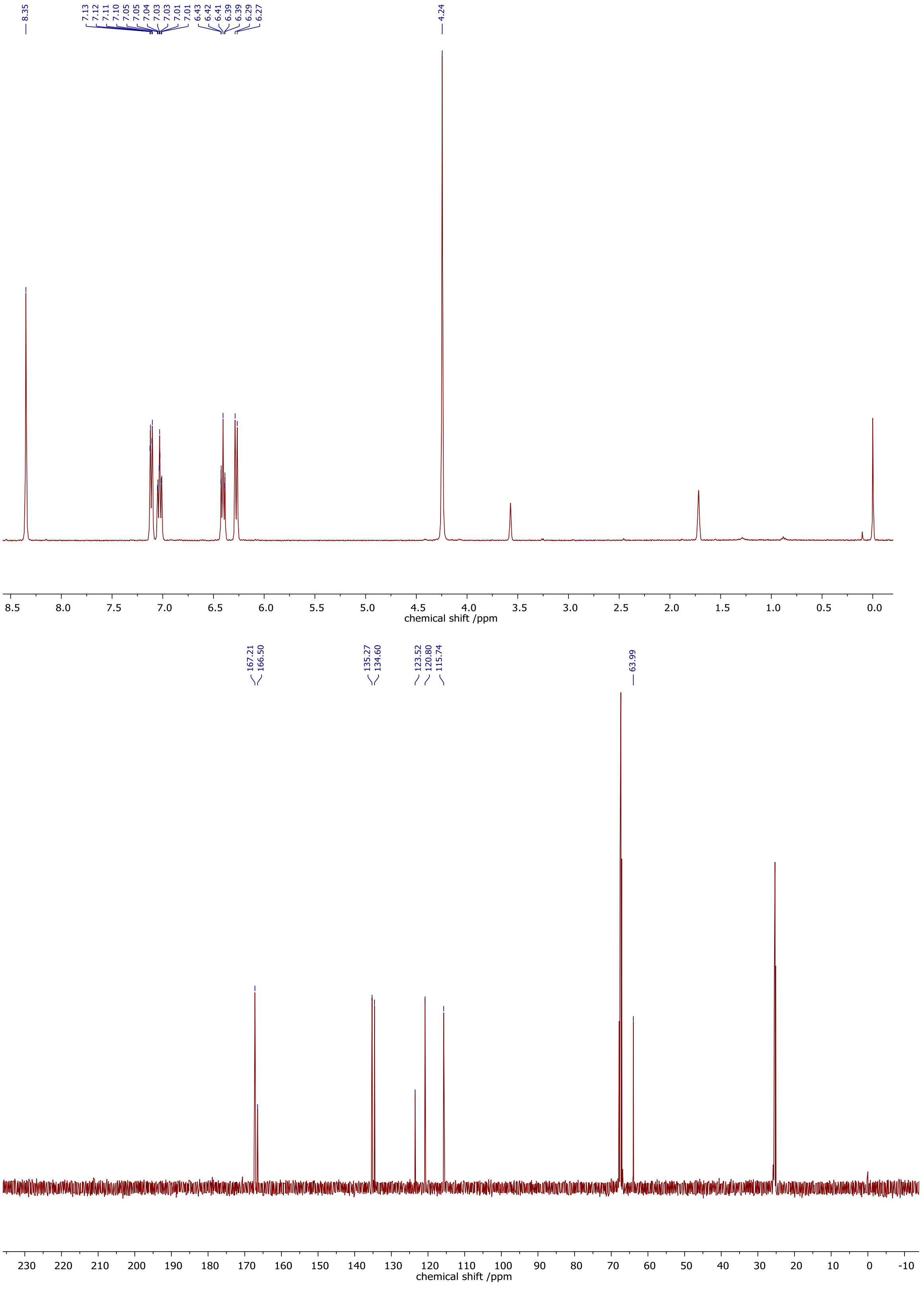

Figure S29. ^{1}H (top) and ^{13}C (bottom) NMR spectra of [Th(salen)$_2$] in thf-d_8.

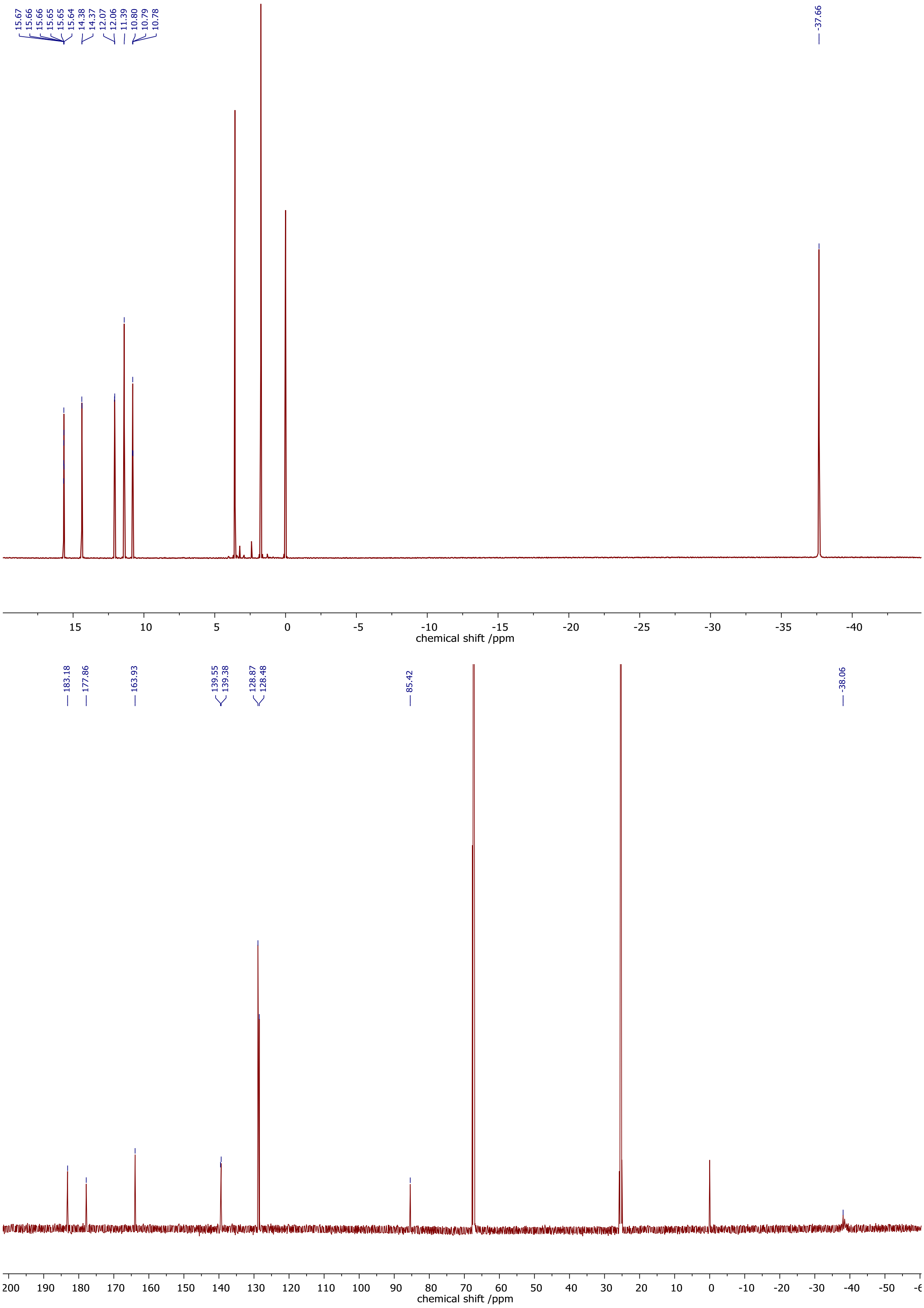

Figure S30. ^{1}H (top) and ^{13}C (bottom) NMR spectra of $[U(salen)_2]$ in thf-d_8.

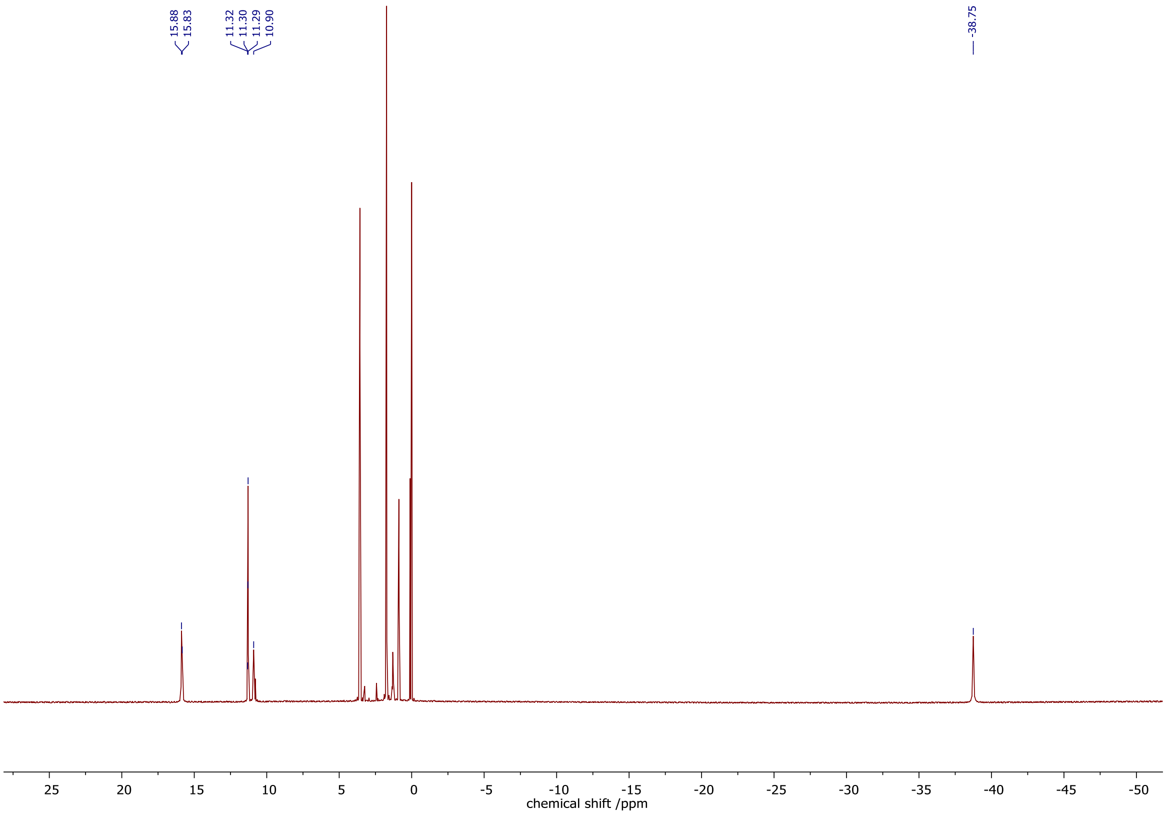

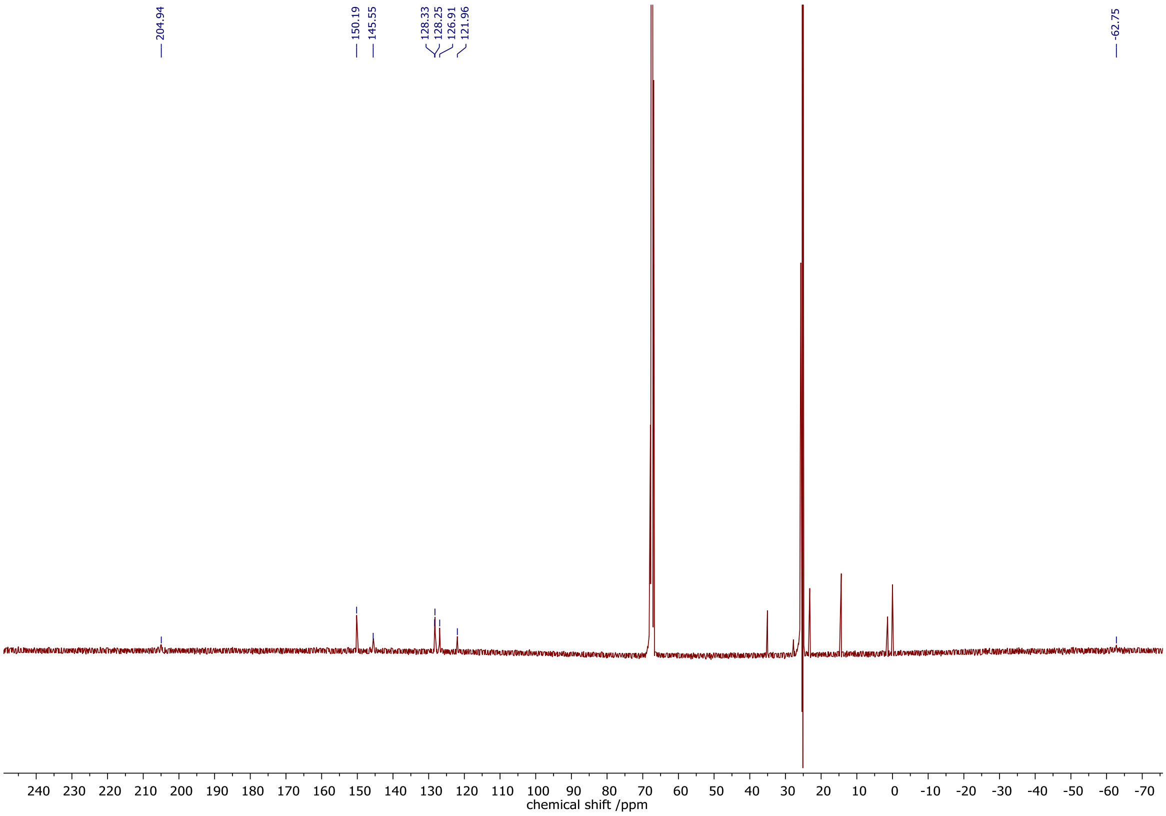

Figure S31. ^{1}H (top) and ^{13}C (bottom) NMR spectra of $[Np(salen)_2]$ in thf-d_8.

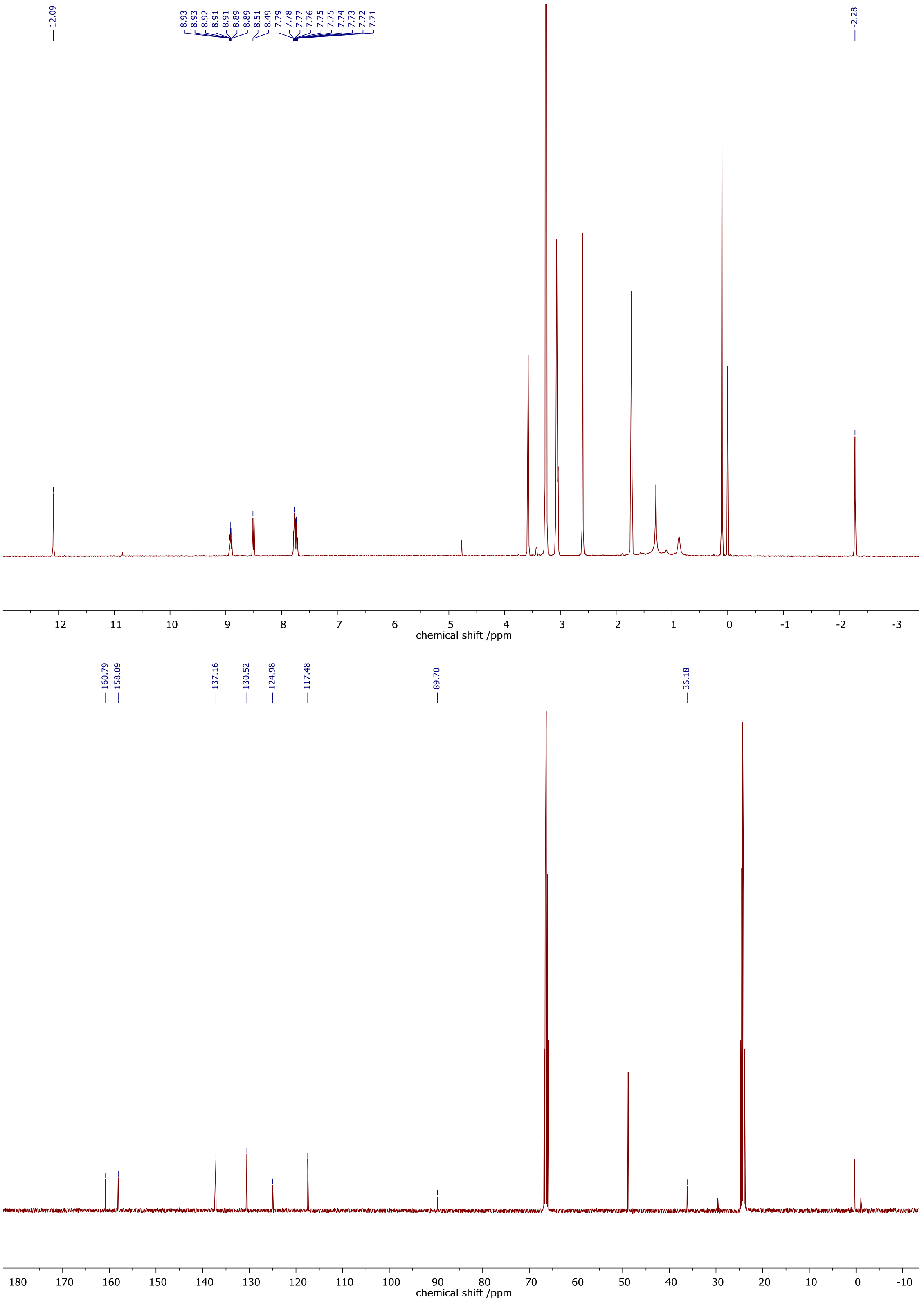

Figure S32. ^{1}H (top) and ^{13}C (bottom) NMR spectra of $[Pu(salen)_2]$ in thf-d_8.

[AnCl$_2$(salen)(MeOH)$_2$]

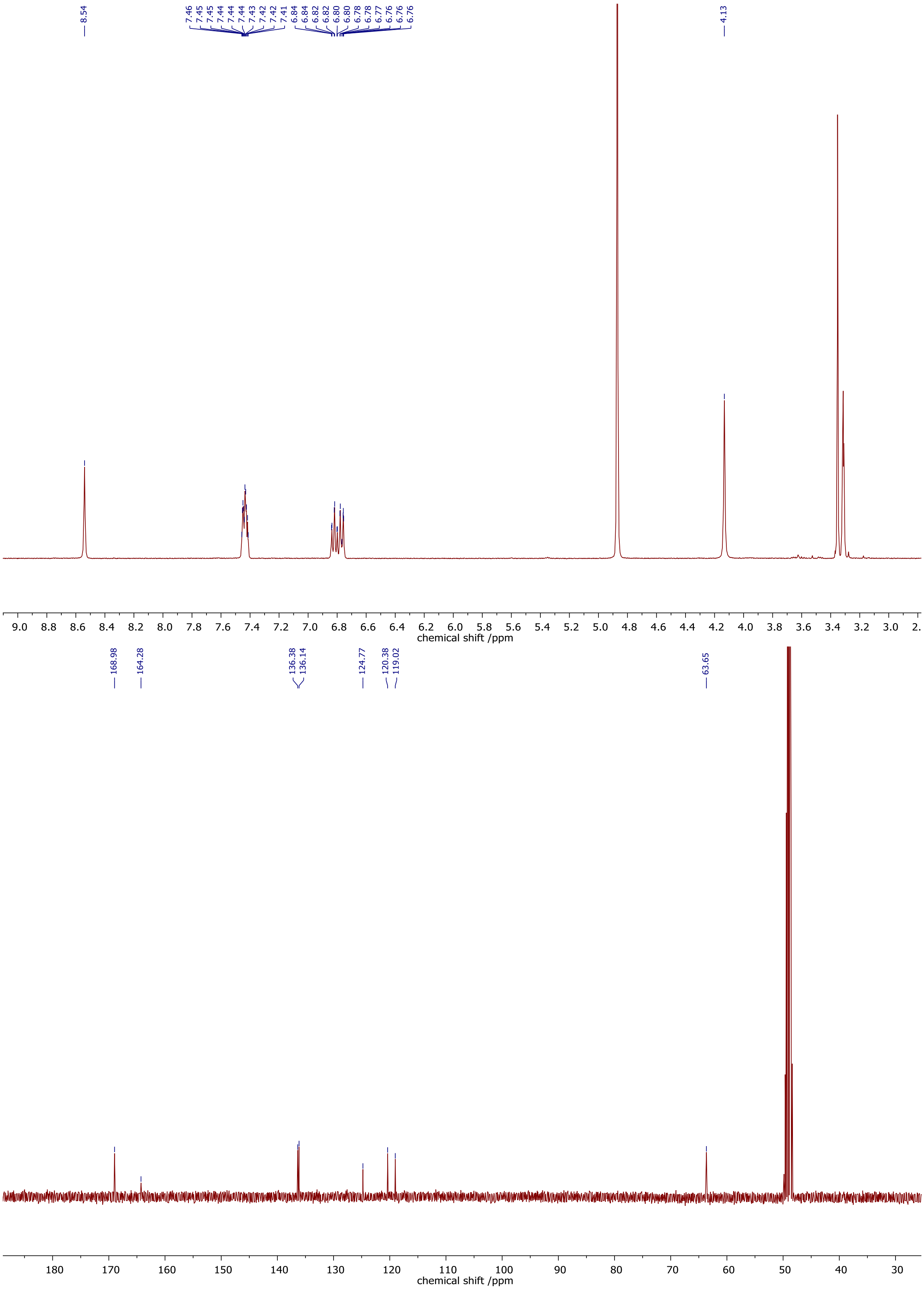

Figure S33. ^{1}H (top) and ^{13}C (bottom) NMR spectra of [ThCl$_2$(salen)(MeOH)$_2$] in MeOD-*d*$_4$.

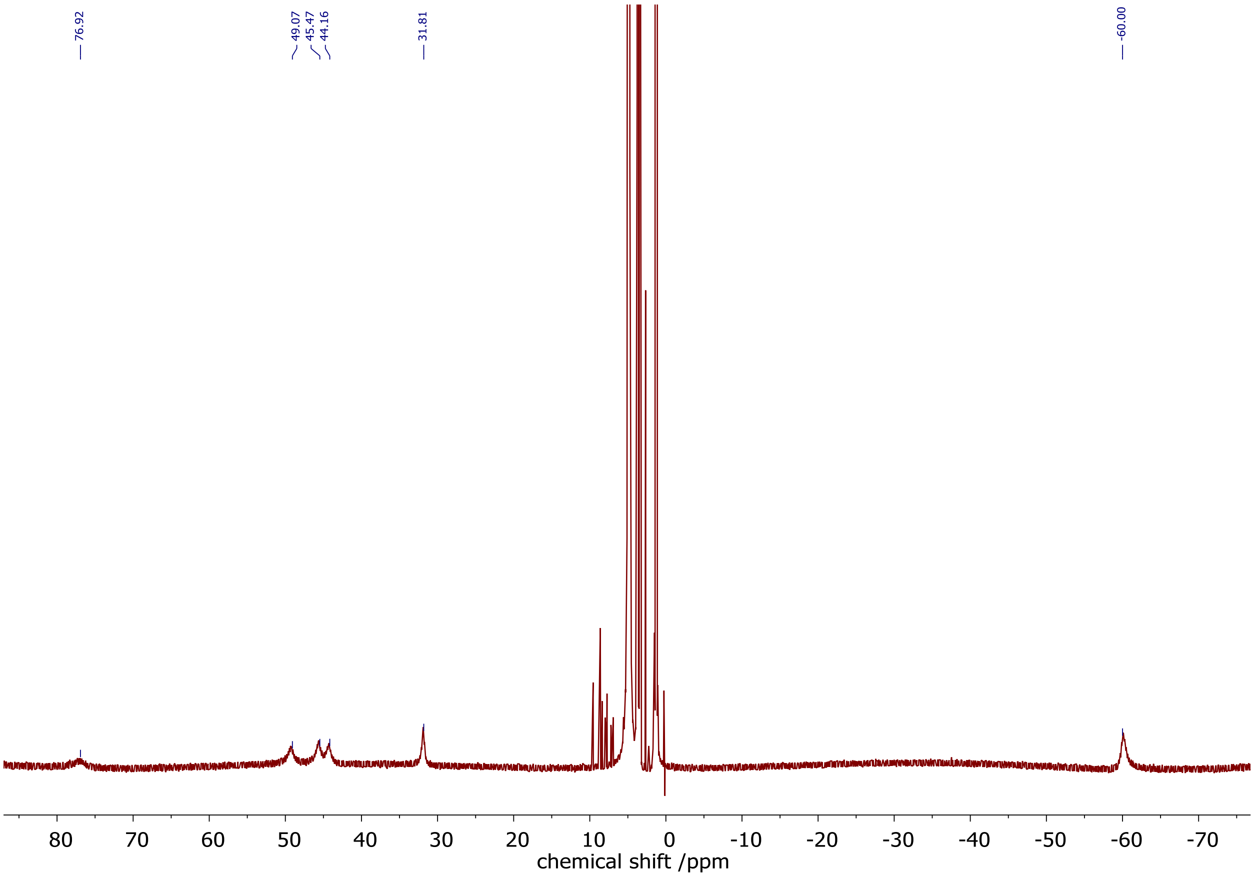

Figure S34. ^{1}H NMR spectrum of $[UCl_2(salen)(MeOH)_2]$ in MeOD-d_4.

[$AnCl_2$(salen)(Py)$_2$]

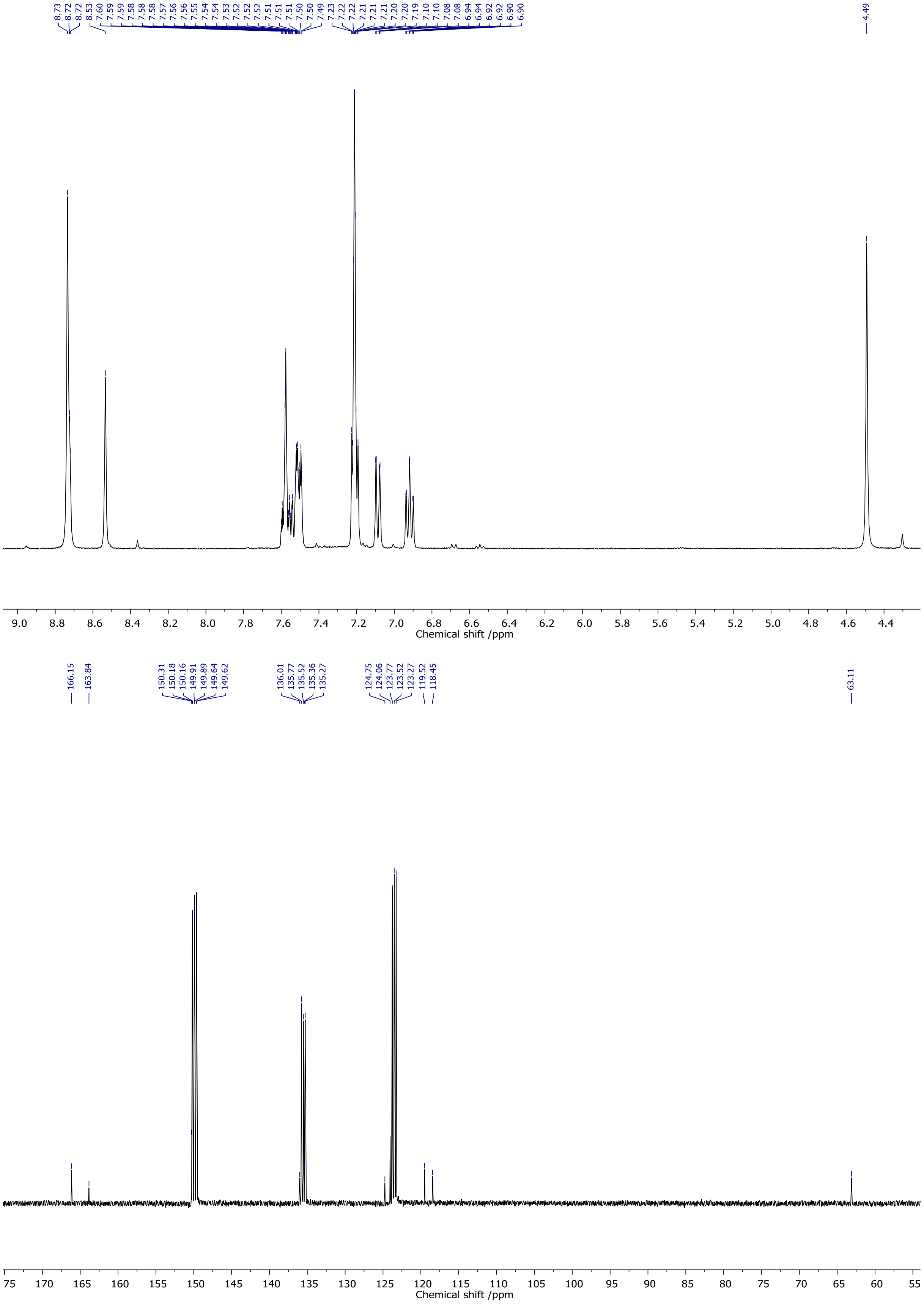

Figure S35. ^{1}H (top) and ^{13}C (bottom) NMR spectra of [$ThCl_2$(salen)(py)$_2$] in Pyridine-*d*$_5$.

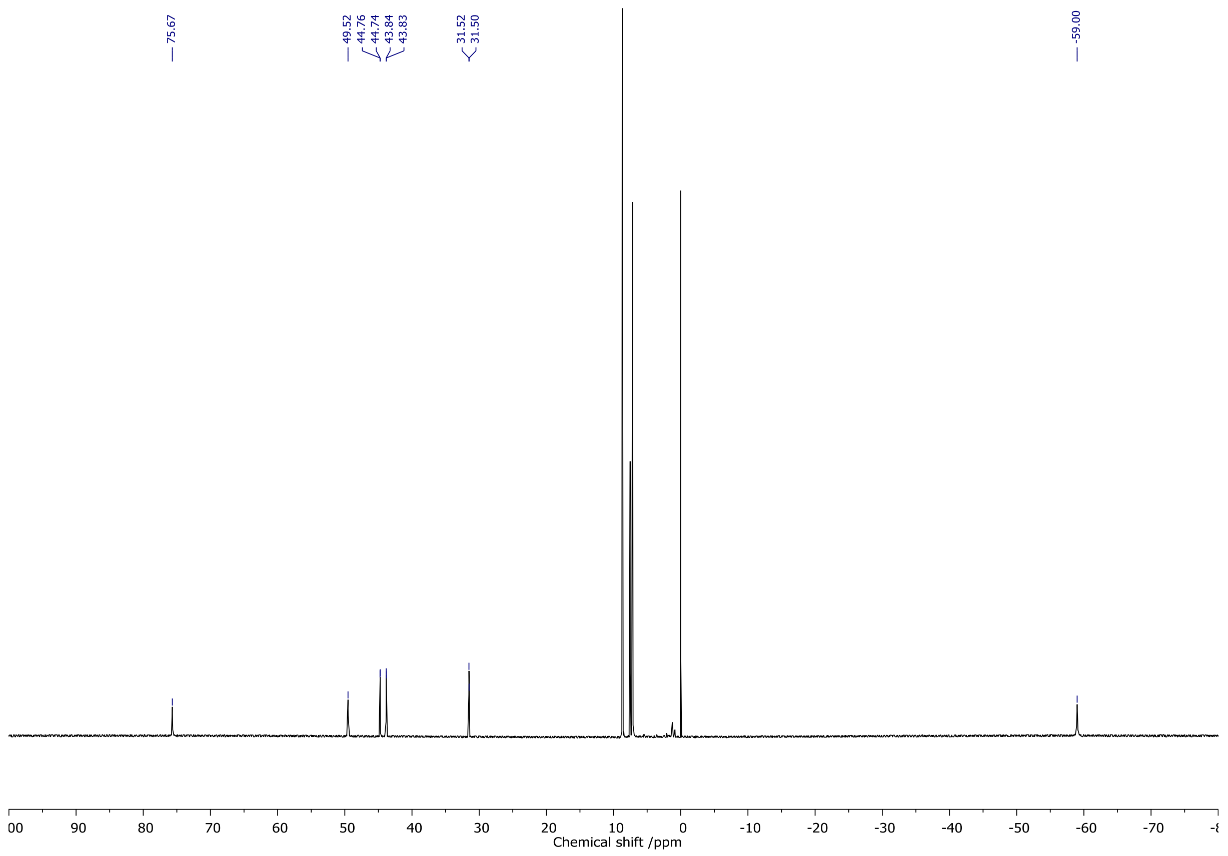

Figure S36. ^{1}H NMR spectrum of $[UCl_2(salen)(py)_2]$ in Pyridine-d_5.

Crystallographic data

Table S16: Crystallographic data of the pyridine complexes [$AnCl_2$(salen)(py)$_2$] (**1**-**4**).

Complex	**1**	**2**	**3**	**4**
Identification code	mo_ThCl2SalenPy2_0m	mo_UCl2salenPy2_0ma	mo_NpCl2SalenPyPy_0m	mo_screening070619_PuClSalenPy_0m
CCDC	1996282	1996277	1996283	1996279
Empirical formula	$C_{31}H_{29}Cl_2N_5O_2Th$	$C_{31}H_{29}Cl_2N_5O_2U$	$C_{31}H_{29}Cl_2N_5NpO_2$	$C_{31}H_{29}Cl_2N_5O_2Pu$
Formula weight	806.53	812.52	811.49	816.49
Temperature/K	100	100	100	100.02
Crystal system	triclinic	triclinic	triclinic	triclinic
Space group	P-1	P-1	P-1	P-1
a/Å	12.9725(8)	12.9818(6)	13.0153(9)	12.9707(10)
b/Å	13.6793(8)	13.6197(6)	13.6124(9)	13.6062(10)
c/Å	17.7662(11)	17.6589(8)	17.7104(12)	17.6525(12)
α/°	78.423(2)	78.2850(10)	77.984(2)	78.044(3)
β/°	80.759(2)	81.561(2)	81.581(2)	81.475(3)
γ/°	89.059(2)	88.880(2)	88.443(2)	88.703(3)
Volume/Å^3	3048.1(3)	3023.9(2)	3035.9(4)	3014.0(4)
Z	4	4	4	4
ρ_{calc}g/cm^3	1.758	1.785	1.775	1.799
μ/mm^{-1}	5.104	5.581	3.636	2.401
F(000)	1560.0	1568.0	1572.0	1576.0
Crystal size/mm^3	0.9 × 0.115 × 0.112	0.151 × 0.087 × 0.077	0.344 × 0.307 × 0.172	0.186 × 0.142 × 0.098
Radiation	MoKα (λ = 0.71073)	MoKα (λ = 0.71073)	MoKα (λ = 0.71073)	MoKα (λ = 0.71073)
2Θ range for data collection/°	4.742 to 52.746	5.324 to 56.71	5.094 to 56.696	4.48 to 61.184
Index ranges	-16 ≤ h ≤ 16,<br>-16 ≤ k ≤ 17,<br>-22 ≤ l ≤ 22	-17 ≤ h ≤ 17,<br>-18 ≤ k ≤ 18,<br>-23 ≤ l ≤ 22	-17 ≤ h ≤ 17,<br>-18 ≤ k ≤ 18,<br>-23 ≤ l ≤ 23	-18 ≤ h ≤ 18,<br>-19 ≤ k ≤ 19,<br>-25 ≤ l ≤ 25
Reflections collected	115990	179401	182038	99027
Independent reflections	12450 [R_{int} = 0.0859,<br>R_{sigma} = 0.0387]	15080 [R_{int} = 0.0631,<br>R_{sigma} = 0.0269]	15138 [R_{int} = 0.0347,<br>R_{sigma} = 0.0156]	18479 [R_{int} = 0.0461,<br>R_{sigma} = 0.0335]
Data/restraints/parameters	12450/48/766	15080/84/776	15138/0/766	18479/0/776
Goodness-of-fit on F^2	1.031	1.097	1.128	1.032
Final R indexes [I>=2σ (I)]	R_1 = 0.0249, wR_2 = 0.0403	R_1 = 0.0220, wR_2 = 0.0490	R_1 = 0.0164, wR_2 = 0.0368	R_1 = 0.0215, wR_2 = 0.0399
Final R indexes [all data]	R_1 = 0.0443, wR_2 = 0.0445	R_1 = 0.0280, wR_2 = 0.0512	R_1 = 0.0205, wR_2 = 0.0382	R_1 = 0.0332, wR_2 = 0.0429
Largest diff. peak/hole / e Å^{-3}	0.83/-0.90	1.01/-1.29	0.60/-0.95	1.02/-1.10

Complex	**5**	**6**	**7**	**8**
Identification code	mo_thcl2salenmepy_0ma_sqd	mo_ucl2salenmepy_0ma_sqd	mo_npcl2salenmepy_2nd_0ma_sqd	pupic_sqd
CCDC	1996281	1996280	1996274	1996276
Empirical formula	$C_{28}H_{28}Cl_2N_4O_2Th$	$C_{28}H_{28}Cl_2N_4O_2U$	$C_{28}H_{28}Cl_2N_4NpO_2$	$C_{28}H_{28}Cl_2N_4O_2Pu$
Formula weight	755.48	761.47	760.44	765.44
Temperature/K	100.0	100.0	100.0	100.03
Crystal system	orthorhombic	orthorhombic	orthorhombic	orthorhombic
Space group	Aea2	Aea2	Aea2	Aea2
a/Å	14.3903(8)	14.3242(14)	14.2883(19)	14.231(3)
b/Å	35.487(2)	35.571(4)	35.540(5)	35.611(6)
c/Å	12.8912(8)	12.8194(14)	12.8033(17)	12.791(2)
Volume/Å^3	6583.2(7)	6531.8(12)	6501.6(15)	6482(2)
Z	8	8	8	8
ρ_{calc}g/cm^3	1.524	1.549	1.554	1.569
μ/mm^{-1}	4.720	5.161	3.389	2.226
F(000)	2912.0	2928.0	2936.0	2944.0
Crystal size/mm^3	0.264 × 0.146 × 0.07	0.495 × 0.255 × 0.108	0.37 × 0.162 × 0.11	0.321 × 0.14 × 0.088
Radiation	MoKα (λ = 0.71073)	MoKα (λ = 0.71073)	MoKα (λ = 0.71073)	MoKα (λ = 0.71073)
2Θ range for data collection/°	5.394 to 54.302	5.392 to 56.66	5.4 to 54.352	5.398 to 51.362
Index ranges	-18 ≤ h ≤ 18, -45 ≤ k ≤ 45, -16 ≤ l ≤ 16	-19 ≤ h ≤ 19, -47 ≤ k ≤ 47, -17 ≤ l ≤ 17	-18 ≤ h ≤ 18, -45 ≤ k ≤ 45, -16 ≤ l ≤ 16	-17 ≤ h ≤ 17, -43 ≤ k ≤ 43, -15 ≤ l ≤ 14
Reflections collected	68637	402637	116575	81567
Independent reflections	7288 [R_{int} = 0.0548, R_{sigma} = 0.0321]	8105 [R_{int} = 0.0469, R_{sigma} = 0.0128]	7169 [R_{int} = 0.0327, R_{sigma} = 0.0129]	5911 [R_{int} = 0.0427, R_{sigma} = 0.0191]
Data/restraints/parameters	7288/1/337	8105/1/337	7169/1/337	5911/1/336
Goodness-of-fit on F^2	1.055	1.075	1.092	1.094
Final R indexes [I>=2σ (I)]	R_1 = 0.0210, wR_2 = 0.0501	R_1 = 0.0218, wR_2 = 0.0503	R_1 = 0.0208, wR_2 = 0.0496	R_1 = 0.0225, wR_2 = 0.0529
Final R indexes [all data]	R_1 = 0.0246, wR_2 = 0.0512	R_1 = 0.0230, wR_2 = 0.0507	R_1 = 0.0216, wR_2 = 0.0500	R_1 = 0.0247, wR_2 = 0.0537
Largest diff. peak/hole / e Å^{-3}	0.45/-0.68	1.53/-1.06	0.50/-1.29	0.95/-0.94
Flack parameter	0.114(7)	0.041(7)	0.144(15)	

Complex	**9**	**10**	**11**	**12**
Identification code	mo_Thlutidin_0m	mo_UCl2SalenLutidin_2nd_0m	mo_NpCl2SalenLutidin_0m	mo_PuCl2salenLutidin_0m
CCDC	1996278	1996275	1996272	1996273
Empirical formula	$C_{33.5}H_{36.5}Cl_2N_{4.5}O_2Th$	$C_{33.5}H_{36.5}Cl_2N_{4.5}O_2U$	$C_{33.5}H_{36.5}Cl_2N_{4.5}NpO_2$	$C_{33.5}H_{36.5}Cl_2N_{4.5}O_2Pu$
Formula weight	837.11	843.10	842.07	847.07
Temperature/K	100.0	100	100.0	99.98
Crystal system	monoclinic	monoclinic	monoclinic	monoclinic
Space group	$P2_1/c$	$P2_1/c$	$P2_1/c$	$P2_1/c$
a/Å	15.4304(12)	15.3984(13)	15.3770(11)	15.3748(11)
b/Å	11.1627(9)	11.1128(9)	11.0989(8)	11.0720(8)
c/Å	20.0575(16)	20.0055(16)	19.9729(15)	19.9925(14)
β/°	104.717(3)	105.196(3)	105.307(2)	105.414(2)
Volume/Å^3	3341.5(5)	3303.6(5)	3287.8(4)	3280.9(4)
Z	4	4	4	4
ρ_{calc}g/cm^3	1.664	1.695	1.701	1.715
μ/mm^{-1}	4.659	5.112	3.360	2.209
F(000)	1636.0	1644.0	1648.0	1652.0
Crystal size/mm^3	0.146 × 0.057 × 0.046	0.166 × 0.066 × 0.045	0.215 × 0.105 × 0.05	0.072 × 0.068 × 0.025
Radiation	MoKα (λ = 0.71073)	MoKα (λ = 0.71073)	MoKα (λ = 0.71073)	MoKα (λ = 0.71073)
2Θ range for data collection/°	4.556 to 54.206	4.388 to 56.684	4.584 to 54.206	5.352 to 50.762
Index ranges	-19 ≤ h ≤ 19, -14 ≤ k ≤ 14, -25 ≤ l ≤ 25	-20 ≤ h ≤ 20, -14 ≤ k ≤ 14, -24 ≤ l ≤ 26	-19 ≤ h ≤ 19, -14 ≤ k ≤ 14, -25 ≤ l ≤ 25	-18 ≤ h ≤ 18, -13 ≤ k ≤ 13, -24 ≤ l ≤ 23
Reflections collected	101838	123138	88448	49163
Independent reflections	7371 [R_{int} = 0.0636, R_{sigma} = 0.0255]	8233 [R_{int} = 0.0387, R_{sigma} = 0.0141]	7257 [R_{int} = 0.0700, R_{sigma} = 0.0309]	6023 [R_{int} = 0.0481, R_{sigma} = 0.0238]
Data/restraints/parameters	7371/54/430	8233/54/430	7257/54/430	6023/54/430
Goodness-of-fit on F^2	1.074	1.149	1.098	1.251
Final R indexes [I>=2σ (I)]	R_1 = 0.0185, wR_2 = 0.0368	R_1 = 0.0173, wR_2 = 0.0345	R_1 = 0.0232, wR_2 = 0.0431	R_1 = 0.0270, wR_2 = 0.0465
Final R indexes [all data]	R_1 = 0.0246, wR_2 = 0.0383	R_1 = 0.0220, wR_2 = 0.0355	R_1 = 0.0320, wR_2 = 0.0453	R_1 = 0.0340, wR_2 = 0.0478
Largest diff. peak/hole / e Å^{-3}	0.40/-0.66	0.43/-0.92	0.44/-0.64	0.79/-1.17

Atomic coordinates x y z for optimised structures

[UCl_2(salen)(py)$_2$]

Energy = -2769.1987871940

U	-0.4486316	0.3392928	0.3035362
Cl	0.0356189	2.9269209	-0.1677476
Cl	-1.9725234	-1.5049575	1.5021685
O	-1.3761862	-0.1803928	-1.6029198
O	1.2310957	0.3096791	1.6944071
N	1.6094333	0.4728101	-1.3588553
N	-2.8048152	1.3524344	0.1774829
N	0.6432953	-2.0230629	-0.1905932
N	-1.1405606	1.4699595	2.4963581
C	2.7621443	1.0135954	-0.9475633
H	2.7578590	1.4631157	0.0490976
C	-2.5095834	-0.4039635	-2.2219068
C	1.7920854	0.9071560	2.7170796
C	-3.7205253	0.1856988	-1.7683095
C	-3.7699016	1.0711295	-0.6217219
H	-4.7547385	1.5392125	-0.4444643
C	3.1755395	0.7699713	2.9477043
H	3.7577614	0.1562528	2.2545083
C	3.7825097	1.4002757	4.0269705
H	4.8597422	1.2813070	4.1815994
C	1.0264801	1.7029450	3.6120861
C	1.5615125	-0.0779747	-2.5767656
H	0.5972178	-0.5025136	-2.8704483
C	-2.5644994	1.7418989	2.5733653
H	-2.7994647	2.4532865	3.3860841
H	-3.0797904	0.7880709	2.7820343
C	-3.0715472	2.2933557	1.2498715
H	-4.1499147	2.5218381	1.3324024
H	-2.5306740	3.2285257	1.0226066
C	3.9137385	1.0160076	-1.7285559
H	4.8323702	1.4658880	-1.3437824
C	1.8962405	-2.3122422	0.1771338
H	2.4146478	-1.5410803	0.7542332
C	-2.5461323	-1.2378442	-3.3572610
H	-1.6097257	-1.6866681	-3.7005022
C	-0.0408559	-2.9317151	-0.8951812
H	-1.0648079	-2.6628180	-1.1679372
C	1.6666082	2.3197271	4.7049237
H	1.0642179	2.9225145	5.3931274
C	3.0309202	2.1808259	4.9175471
H	3.5121830	2.6712818	5.7678509
C	-4.9161824	-0.0715372	-2.4671745
H	-5.8414610	0.3960842	-2.1134704
C	-0.4034409	1.8875937	3.4616377
H	-0.8816842	2.4496541	4.2836573
C	-4.9397488	-0.9008375	-3.5797252
H	-5.8776856	-1.0945459	-4.1069300
C	3.8637305	0.4370394	-2.9962556
H	4.7502160	0.4197930	-3.6372338
C	2.6605216	-0.1164354	-3.4304355
H	2.5681969	-0.5775762	-4.4168735
C	-3.7423695	-1.4839410	-4.0198760
H	-3.7451738	-2.1391174	-4.8969333
C	0.4994078	-4.1585823	-1.2680297
H	-0.1007415	-4.8649075	-1.8470492
C	1.8078310	-4.4563875	-0.8887494
H	2.2658343	-5.4104913	-1.1660950
C	2.5188578	-3.5143846	-0.1472659
H	3.5455484	-3.7011650	0.177138

$[UCl_2(salen)(pic)_2]$

Energy = -2847.6802872100

U	0.3811981	-0.6996830	0.8470910
Cl	3.0404585	-0.8128170	0.5688297
Cl	-1.7868847	-1.4643989	2.2217757
O	0.2625282	1.2555629	1.8075961
C	1.0589267	-3.2985852	2.9462327
H	1.5377478	-4.2625455	3.1983131
H	0.0438665	-3.2862135	3.3798239
O	0.1873288	-2.0119461	-0.8887764
C	0.1357654	1.8868635	2.9485741
N	0.9326088	-3.1236611	1.5111576
N	1.2315917	-0.8832013	3.2591300
C	-0.3877041	3.1951102	2.9817414
H	-0.6787989	3.6605240	2.0357823
C	0.5190030	1.2741966	4.1727716
C	1.8637820	-2.1573739	3.5484020
H	1.9767807	-2.3177240	4.6362664
H	2.8675928	-2.1486198	3.0888951
C	1.1935620	-4.1349429	0.7640019
H	1.5175180	-5.0758064	1.2441482
N	-1.7300017	0.3244239	-0.3594231
N	1.0117031	0.9327386	-1.1283290
C	1.0871442	-0.0582166	4.2326933
H	1.4267324	-0.3660454	5.2378903
C	1.5835444	0.4529527	-2.2376265
H	1.8280891	-0.6126601	-2.2361347
C	0.7100833	2.2364651	-1.0945549
H	0.2474790	2.5951596	-0.1707747
C	-0.5313287	3.8735818	4.1856501
H	-0.9443243	4.8874659	4.1844271
C	-2.0555593	-0.0092875	-1.6144618
H	-1.4347468	-0.7806675	-2.0789436
C	1.1099623	-4.1937697	-0.6817216
C	-2.4658888	1.2602114	0.2490397
H	-2.1869071	1.5036353	1.2779335
C	-3.1128309	0.5729084	-2.3010578
H	-3.3316098	0.2518944	-3.3234994
C	1.8666786	1.2437700	-3.3455773
H	2.3354338	0.7910866	-4.2238409
C	1.5511701	2.6071676	-3.3254149
C	0.5352024	-3.2640411	-2.8577499
H	0.1395202	-2.4323003	-3.4474838
C	0.9609328	3.0988086	-2.1536270
H	0.6931473	4.1552717	-2.0620082
C	-3.8826300	1.5658424	-1.6805920
C	0.3720558	1.9903591	5.3769086
H	0.6786673	1.5105920	6.3126883
C	-3.5338144	1.9023614	-0.3677148
H	-4.0935123	2.6644019	0.1817884
C	1.5301562	-5.3720470	-1.3291268
H	1.9143486	-6.1969269	-0.7193186
C	0.9682687	-4.4333450	-3.4707305
H	0.9133199	-4.5201226	-4.5606873
C	1.4694675	-5.4998323	-2.7096946
H	1.8053118	-6.4192743	-3.1965654
C	-0.1511674	3.2755373	5.3960954
H	-0.2628138	3.8146423	6.3406076
C	1.8127720	3.4996827	-4.4992784
H	2.4742726	3.0171128	-5.2359511
H	2.2697696	4.4510757	-4.1785223
H	0.8629804	3.7500122	-5.0060652
C	0.5978874	-3.1185090	-1.4572215
C	-5.0150539	2.2332312	-2.3985216
H	-4.6313454	2.8433636	-3.2359075
H	-5.5888100	2.8919645	-1.7278688
H	-5.7006278	1.4845860	-2.8314834

[UCl_2(salen)(lut)$_2$]

Energy = -2926.1590157660

U	1.4482815	-0.0030929	-0.0122186
Cl	2.1465831	2.4076038	-0.9251977
Cl	2.1903832	-2.3891769	0.9399201
O	0.9351249	-0.5762299	-2.0578085
N	-0.8029589	1.3748229	-0.3250965
O	0.9113222	0.5735112	2.0212775
N	3.5729245	0.5149574	1.3226822
C	-1.1652913	-1.6993354	1.5588493
H	-0.3915123	-1.6461974	2.3306727
C	-1.2144278	1.6469854	-1.5659980
H	-0.4627151	1.5580062	-2.3561496
C	2.1236839	0.5289708	5.9772162
H	2.4380019	0.5190213	7.0242384
C	-2.4848692	-2.0068881	1.8960433
C	-1.6679617	-1.5134423	-0.6767548
H	-1.2898251	-1.2976452	-1.6805046
C	0.7615302	0.5108719	5.6431369
H	0.0060519	0.4851249	6.4349689
C	2.1694557	-0.5841114	-6.0083051
H	2.4894214	-0.5874173	-7.0536535
N	-0.7723534	-1.4356138	0.3096958
C	-2.8840845	-2.2617565	3.3193270
H	-3.2707748	-3.2883264	3.4454324
H	-3.6855523	-1.5697375	3.6320973
H	-2.0305237	-2.1312496	4.0043219
C	1.3341370	-0.5753613	-3.3060701
C	-3.4149829	-2.0410129	0.8519602
H	-4.4671906	-2.2563346	1.0703522
C	1.3030079	0.5553674	3.2715675
C	3.1073288	-0.5836569	-4.9854374
H	4.1766170	-0.5893584	-5.2228961
C	4.7637773	0.5910465	0.4967432
H	5.6849831	0.5560433	1.1065475
H	4.7394418	1.5506895	-0.0484106
C	0.3554517	0.5291720	4.3144269
H	-0.7063836	0.5215329	4.0519047
C	4.7754137	-0.5470992	-0.5122149
H	5.7006708	-0.4995278	-1.1150353
H	4.7606420	-1.5065507	0.0336542
N	3.5896203	-0.4895871	-1.3473897
C	2.6846772	0.5663001	3.6039647
C	3.7526602	-0.5824528	-2.6179319
H	4.7800841	-0.6829585	-3.0112329
C	3.7252283	0.6028195	2.5951976
H	4.7483956	0.7157587	2.9958540
C	-1.6749943	1.4823890	0.6800510
H	-1.2828069	1.2547097	1.6752082
C	-3.0171393	1.8194182	0.4986193
C	-2.5294616	2.0031518	-1.8745382
C	0.8054272	-0.5842811	-5.6813433
H	0.0540416	-0.5874067	-6.4774680
C	-3.4343529	2.0726854	-0.8109360
H	-4.4829419	2.3239864	-1.0071657
C	-3.0175206	-1.8034730	-0.4663761
C	0.3923463	-0.5844649	-4.3546032
H	-0.6709468	-0.5912168	-4.0977109
C	-2.9471984	2.2836079	-3.2877758
H	-3.1053711	3.3658812	-3.4444830
H	-3.8968492	1.7753793	-3.5268516
H	-2.1824300	1.9477178	-4.0072200
C	3.0669517	0.5623212	4.9596833
H	4.1346126	0.5812280	5.2036969
C	-3.9671407	1.8570723	1.6583761
H	-3.4349732	1.7302896	2.6152665
H	-4.7117405	1.0450487	1.5753330
H	-4.5222660	2.8101081	1.6929474
C	2.7173917	-0.5716866	-3.6320162
C	-3.9930269	-1.7974057	-1.6054087
H	-4.5944824	-0.8699661	-1.5900642
H	-4.6939397	-2.6464674	-1.5418309
H	-3.4746825	-1.8431116	-2.5771369

[$ThCl_2$(salen)(pic)$_2$]

Energy = -2778.4743439800

Th	0.4326158	-0.6726898	-0.8065539
Cl	3.1483545	-0.7590574	-0.5132236
Cl	-1.7632186	-1.5145462	-2.2026912
O	0.2611554	1.3082365	-1.8648377
N	1.0158579	-3.1327977	-1.5067371
O	0.2322251	-2.0568988	0.9596294
N	-1.7172068	0.3452252	0.4508108
N	1.0391662	1.0394737	1.1725178
N	1.3215818	-0.8737475	-3.2634900
C	0.5804200	-3.2032525	1.4844947
C	0.1420863	1.8975196	-3.0261306
C	-0.5550530	3.8255610	-4.3426607
H	-0.9958378	4.8270246	-4.3818193
C	-0.4143780	3.1906770	-3.1153154
H	-0.7357706	3.6768038	-2.1895890
C	-3.9087140	1.5312723	1.7534843
C	-2.0208354	0.0294449	1.7149763
H	-1.3701612	-0.7080328	2.1949271
C	-3.5810494	1.8499987	0.4290153
H	-4.1760890	2.5726639	-0.1363379
C	0.4565242	-3.4184400	2.8729840
H	0.0653223	-2.6020191	3.4869940
C	1.2281322	-4.1601784	-0.7643507
H	1.5528889	-5.0970564	-1.2519525
C	0.8067040	2.3527312	1.0671435
H	0.4181122	2.6951074	0.1031050
C	1.0389061	3.2452008	2.1067032
H	0.8329329	4.3088640	1.9584494
C	1.4401397	-5.4829524	1.2837137
H	1.8211006	-6.2913265	0.6501225
C	1.9824341	-2.1396106	-3.5265661
H	2.9747898	-2.1117877	-3.0434023
H	2.1253693	-2.3053900	-4.6101945

C	1.1687939	-0.0624959	-4.2484495
H	1.5288998	-0.3778271	-5.2443955
C	0.5673995	1.2576166	-4.2254106
C	1.3181746	-5.6767172	2.6520641
H	1.6018929	-6.6304436	3.1049436
C	1.0847391	-4.2616516	0.6759437
C	0.8211357	-4.6309247	3.4441214
H	0.7155285	-4.7686703	4.5251558
C	-0.1371566	3.1996935	-5.5266165
H	-0.2474921	3.7036722	-6.4904813
C	1.7786926	1.3977140	3.4225202
H	2.1729631	0.9610017	4.3443651
C	1.7588096	3.6894824	4.4922389
H	1.9120807	4.7296323	4.1629701
H	2.6273771	3.3691961	5.0906272
H	0.8768722	3.6758968	5.1590490
C	1.5313789	2.7739898	3.3295645
C	0.4219920	1.9319976	-5.4546793
H	0.7586498	1.4315129	-6.3691200
C	1.5226167	0.5768443	2.3322021
H	1.7106954	-0.4996940	2.3854179
C	-2.4913504	1.2386954	-0.1772718
H	-2.2239978	1.4707210	-1.2123890
C	-3.0964263	0.5880010	2.3945925
H	-3.2978699	0.2841006	3.4255662
C	1.1818096	-3.2939866	-2.9407664
H	0.1770711	-3.2976033	-3.3988397
H	1.6808404	-4.2493799	-3.1864430
C	-5.0563203	2.1876287	2.4563031
H	-4.7193385	3.1291442	2.9283240
H	-5.4619690	1.5437550	3.2532221
H	-5.8649978	2.4430774	1.7524526

[$PaCl_2$(salen)(pic)$_2$]

Energy = -2812.1553271250

Pa	0.3946202	-0.6728057	0.8214948
Cl	3.0753366	-0.7181427	0.5542298
Cl	-1.8026773	-1.4968715	2.1449208
O	0.2245025	1.2814781	1.8452576
C	1.1107159	-3.2788277	2.9376605
H	1.6013901	-4.2377463	3.1853244
H	0.0978856	-3.2802045	3.3772704
O	0.2345670	-2.0169903	-0.9248024
C	0.1181246	1.9044349	2.9924171
N	0.9735096	-3.1039272	1.5024023
N	1.2446446	-0.8653589	3.2578363
C	-0.3992237	3.2148415	3.0488730
H	-0.6990130	3.6956428	2.1133083
C	0.5243857	1.2799356	4.2035632
C	1.9036605	-2.1292228	3.5358763
H	2.0312047	-2.2909683	4.6217627
H	2.9024383	-2.1015962	3.0661379
C	1.2077301	-4.1290719	0.7616198
H	1.5258700	-5.0663488	1.2519288
N	-1.7144955	0.3270211	-0.4615025
N	1.0123290	1.0227901	-1.1364364
C	1.1031725	-0.0473745	4.2401015
H	1.4624806	-0.3602693	5.2366390
C	1.3306053	0.5641698	-2.3520138
H	1.3294964	-0.5224259	-2.4736627
C	1.0123535	2.3487279	-0.9490956
H	0.7538764	2.6890205	0.0576445
C	-0.5249198	3.8792107	4.2623184
H	-0.9357036	4.8938729	4.2785421
C	-2.2129156	-0.2577971	-1.5586432
H	-1.6962306	-1.1608047	-1.8954850
C	1.0909657	-4.2193122	-0.6793431
C	-2.3187452	1.4335876	-0.0157641
H	-1.8944645	1.8842461	0.8855869
C	-3.3157711	0.2357112	-2.2427342
H	-3.6766705	-0.2909645	-3.1308027
C	1.6467393	1.3995637	-3.4169654
H	1.8974339	0.9622481	-4.3874411
C	1.6426503	2.7875886	-3.2369405
C	0.4709268	-3.3412141	-2.8651587
H	0.0740931	-2.5197875	-3.4685312
C	1.3203118	3.2542127	-1.9559415
H	1.3061459	4.3257808	-1.7374183
C	-3.9515035	1.4002871	-1.7919863
C	0.3957497	1.9828152	5.4183667
H	0.7187637	1.4932541	6.3435193
C	-3.4235048	1.9985908	-0.6425687
H	-3.8705648	2.9069173	-0.2291146
C	1.4606872	-5.4273879	-1.3039725
H	1.8416277	-6.2441975	-0.6813462
C	0.8508380	-4.5404896	-3.4544159
H	0.7544230	-4.6602373	-4.5383651
C	1.3518436	-5.5955581	-2.6768323
H	1.6479468	-6.5379661	-3.1452461
C	-0.1288480	3.2663986	5.4606821
H	-0.2273293	3.7942005	6.4130122
C	1.9455031	3.7331708	-4.3578968
H	2.4387792	3.2204325	-5.1987635
H	2.5887043	4.5613928	-4.0162952
H	1.0092668	4.1825345	-4.7367802
C	0.5876685	-3.1524197	-1.4728881
C	-5.1279330	1.9750468	-2.5188270
H	-4.8111622	2.3812616	-3.4964290
H	-5.6004344	2.7887519	-1.9465691
H	-5.8838575	1.1971495	-2.7213180

[$NpCl_2$(salen)(pic)$_2$]

Energy = -2885.1650553430

Np	-0.3766856	0.7119722	-0.8543559
Cl	-3.0227501	0.9869186	-0.5450927
Cl	1.7558311	1.4361931	-2.3004195
O	-0.3153236	-1.2518167	-1.7882497
N	1.7055879	-0.3200407	0.3270151
N	-0.9074464	3.1202286	-1.5349191
N	-1.0514380	-0.8809381	1.1017677
O	-0.1105006	2.0247530	0.8590987
N	-1.2138791	0.8924515	-3.2660177
C	-1.0296930	3.3039551	-2.9683983
H	-0.0152753	3.2884615	-3.4023040
H	-1.5064112	4.2699821	-3.2159802
C	-0.5999277	3.0942768	1.4378618
C	-0.1225835	-1.8719358	-2.9263439
C	-1.1992752	4.1158457	-0.7798978
H	-1.5344512	5.0556296	-1.2539622
C	0.4245400	-3.1706174	-2.9446132
H	0.6855505	-3.6365323	-1.9901832
C	0.6267330	-3.8400738	-4.1455339
H	1.0564300	-4.8469381	-4.1325994
C	-0.5794137	3.2165444	2.8415179
H	-0.1556912	2.3957925	3.4271466
C	-0.8950448	-2.9971976	2.2157919
H	-0.5955994	-4.0474606	2.1597518
C	-1.6078110	-0.3719202	2.2071635
H	-1.8968091	0.6814020	2.1653470
C	-1.4553152	-2.4717572	3.3871730
C	3.7842174	-1.6908926	1.6366970
C	-0.7102608	-2.1756821	1.1118399
H	-0.2661712	-2.5593892	0.1891668
C	-1.6191208	5.4061929	2.7087195
H	-2.0113860	6.2993068	3.2023509
C	-0.4664090	-1.2596385	-4.1617952
C	-1.6386490	5.3009255	1.3244967
H	-2.0472322	6.1174717	0.7193377
C	-1.8394690	2.1657963	-3.5697042
H	-2.8455745	2.1670151	-3.1157034
H	-1.9437628	2.3183292	-4.6594696
C	3.4889960	-1.9196067	0.2873919
H	4.0635454	-2.6440310	-0.2963914
C	-1.0492389	0.0659478	-4.2340948
H	-1.3740756	0.3691771	-5.2454308
C	1.9847425	-0.0837007	1.6150716
H	1.3563080	0.6616884	2.1103160
C	-1.0858768	4.3516437	3.4635539
H	-1.0624856	4.4203308	4.5559245
C	-1.8231092	-1.1211143	3.3573736
H	-2.2785714	-0.6455217	4.2303905
C	3.0031852	-0.7345583	2.2984471
H	3.1828393	-0.4944975	3.3501023
C	0.2836565	-3.2427877	-5.3670587
H	0.4412656	-3.7750247	-6.3089022
C	-0.2626763	-1.9667278	-5.3623425
H	-0.5413746	-1.4873652	-6.3070286
C	-1.1480580	4.1558606	0.6687101
C	2.4551374	-1.2189420	-0.3215416
H	2.2181139	-1.3798056	-1.3764960
C	-1.6272489	-3.3106285	4.6152687
H	-1.9341729	-4.3377736	4.3576254
H	-2.3697501	-2.8750865	5.3026351
H	-0.6670023	-3.3824986	5.1586454
C	4.8658836	-2.4429759	2.3481014
H	4.4235066	-3.2438477	2.9686792
H	5.4314001	-1.7812757	3.0250960
H	5.5655307	-2.9134920	1.6392535

[$PuCl_2$(salen)(pic)$_2$]

Energy = -2924.6207224360

Pu	-0.3129358	0.7043178	-0.8697425
Cl	-2.9406043	0.9611925	-0.4616942
Cl	1.7979200	1.3523945	-2.3225057
C	-1.1718008	4.0904622	-0.8165435
H	-1.5325367	5.0160854	-1.2991695
C	-1.0600562	3.2480233	-2.9932895
H	-0.0669897	3.1949419	-3.4721707
H	-1.5207851	4.2218854	-3.2399108
C	-0.5144953	3.1112143	1.3988706
O	-0.0391942	2.0333022	0.8306150
N	-0.8828404	3.0867107	-1.5632407
C	-1.0876921	4.1578140	0.6270572
O	-0.2183622	-1.2342594	-1.8161452
C	-0.1006554	-1.8775234	-2.9500739
N	-1.2911045	0.8473844	-3.2347083
C	0.4687662	-3.1658915	-2.9851470
H	0.8094506	-3.6095573	-2.0454150
C	-0.5508971	-1.2949907	-4.1642336
C	-1.1572755	0.0204338	-4.2079425
H	-1.5317751	0.3174251	-5.2037652
C	-1.9256103	2.1179469	-3.5256006
H	-2.8993062	2.1408616	-3.0067566
H	-2.0947394	2.2523319	-4.6095248
N	-0.9872610	-0.8625769	1.0973640
N	1.7456654	-0.3213335	0.4104121
C	-0.7701097	-2.1823279	1.0495792
H	-0.3636593	-2.5648165	0.1093317
C	-1.5709741	5.3110998	1.2745028
H	-2.0020751	6.1130621	0.6655109
C	0.5925253	-3.8528658	-4.1868352
H	1.0413532	-4.8513719	-4.1901803
C	-0.9516347	4.4052948	3.4141930
H	-0.8981132	4.4950525	4.5039841
C	-0.4552935	3.2622118	2.8001662
H	-0.0105015	2.4560237	3.3903225
C	2.4731221	-1.2695509	-0.1906847
H	2.2029498	-1.5042651	-1.2238238
C	-1.5154599	5.4415368	2.6550900
H	-1.9021932	6.3400356	3.1432697
C	-1.4894309	-0.3514834	2.2263089
H	-1.6753176	0.7252101	2.2296911
C	-0.4212328	-2.0172585	-5.3656660
H	-0.7773439	-1.5595941	-6.2948982
C	-1.5513730	-2.5077824	3.3090711
C	-1.0393297	-3.0315056	2.1150687
H	-0.8463051	-4.1028727	2.0100361
C	2.0592458	0.0038267	1.6693520
H	1.4452630	0.7869398	2.1227034
C	0.1481773	-3.2831402	-5.3889171
H	0.2464226	-3.8292231	-6.3308956
C	3.0951945	-0.6003911	2.3703658
H	3.3058445	-0.2855371	3.3963256
C	3.8560488	-1.6045156	1.7580864
C	3.5216509	-1.9301563	0.4379537
H	4.0782777	-2.6973723	-0.1075094
C	-1.7782218	-1.1268126	3.3429645
H	-2.1853852	-0.6484253	4.2380959
C	-1.8220814	-3.3838529	4.4930575
H	-2.2757858	-4.3404352	4.1845193
H	-2.4871878	-2.8895435	5.2188136
H	-0.8752062	-3.6235376	5.0108797
C	4.9564545	-2.3085317	2.4901729
H	5.5012021	-1.6168851	3.1537866
H	5.6708562	-2.7758304	1.7937444
H	4.5347424	-3.1090037	3.125724

[ThCl$_2$(salen)(pic)$_2$] twisted (please also see Figure S15)

Th	-0.3644887	0.0561317	-0.0304472
Cl	0.5592821	1.7490808	1.9287923
Cl	-2.4953920	-0.8941124	-1.4554248
O	-0.6255874	1.9686502	-1.1791752
N	0.3388032	-2.4111250	-0.7540892
O	-0.5335093	-1.4047861	1.6750587
N	-2.5427546	0.9906045	1.1879151
N	2.2876148	-0.0695869	0.0212720
N	0.6200723	-0.1418936	-2.4935491
C	-0.2273473	-2.5610489	2.1976147
C	-0.7522136	2.5375074	-2.3455713
C	-1.5341331	4.3980336	-3.7084643
H	-2.0388280	5.3673669	-3.7763887
C	-1.3909549	3.7901834	-2.4683232
H	-1.7693093	4.2636346	-1.5579564
C	-4.7980004	2.0712510	2.4710101
C	-2.8312384	0.6726056	2.4549658
H	-2.1428391	-0.0236483	2.9432935
C	-4.4825788	2.3946350	1.1446842
H	-5.1137253	3.0783416	0.5699766
C	-0.4095342	-2.8009362	3.5761468
H	-0.8166743	-1.9923221	4.1897537
C	0.4935205	-3.4694804	-0.0385269
H	0.8086897	-4.3998032	-0.5443522
C	2.9852783	0.9198864	-0.5491442
H	2.4122466	1.6706951	-1.1036215
C	4.3679257	1.0110396	-0.4648325
H	4.8836654	1.8388367	-0.9588948
C	0.6256147	-4.8450510	1.9862065
H	1.0255452	-5.6434686	1.3517569
C	1.3202817	-1.3880673	-2.7496727
H	2.3088465	-1.3495428	-2.2591950
H	1.4861386	-1.5454044	-3.8310485
C	0.4254948	0.6336445	-3.5023589
H	0.8042973	0.3085204	-4.4880706
C	-0.2485523	1.9147318	-3.5226495
C	0.4501733	-5.0611168	3.3443981
H	0.7116671	-6.0237469	3.7917265
C	0.3006976	-3.6086802	1.3907765
C	-0.0728998	-4.0259769	4.1359041
H	-0.2194557	-4.1838860	5.2094206
C	-1.0385907	3.7876512	-4.8716911
H	-1.1527267	4.2724194	-5.8449274
C	4.3407862	-0.9859599	0.8429120
H	4.8340058	-1.7797662	1.4104142
C	6.5734352	0.1105712	0.3891355
H	7.0313728	0.6747438	-0.4389536
H	7.0199467	-0.8962136	0.4306266
H	6.8357893	0.6270374	1.3310042
C	5.0851086	0.0402387	0.2468324
C	-0.3997609	2.5612527	-4.7665074
H	-0.0037663	2.0720229	-5.6631731
C	2.9586136	-1.0003168	0.7084974
H	2.3657033	-1.7959251	1.1709901
C	-3.3591641	1.8366938	0.5483785
H	-3.0984253	2.0725071	-0.4873721
C	-3.9377549	1.1807241	3.1251728
H	-4.1272696	0.8773845	4.1586437
C	0.5318341	-2.5578218	-2.1867126
H	-0.4650998	-2.5724415	-2.6617398
H	1.0477139	-3.5042427	-2.4318714
C	-5.9841677	2.6697309	3.1617461
H	-5.7051220	3.6366574	3.6199128
H	-6.3538344	2.0151737	3.9674427
H	-6.8044661	2.8661823	2.4523235

[$ThCl_2$(salen)(pic)$_2$] double twisted (please also see Figure S16)

Th	-0.4981168	0.9001783	1.0462106
Cl	0.5346161	2.8014355	2.7177007
Cl	-2.8946491	1.4435488	2.2414696
O	-0.9024567	2.6878344	-0.2465553
N	0.1237899	-1.6207142	0.2930698
O	-0.3082313	-0.5143273	2.7759872
N	-2.4348651	-0.2343231	-0.3996105
N	2.1635896	0.7331255	0.9752032
N	0.4435134	0.6227891	-1.4716780
C	-0.1099103	-1.6950983	3.2877310
C	-0.8231357	3.3455627	-1.3674940
C	-1.2890805	5.3323579	-2.6911192
H	-1.7165341	6.3379895	-2.7611497
C	-1.3636646	4.6436713	-1.4883833
H	-1.8421895	5.0834054	-0.6090953
C	-4.5973147	-1.3910931	-1.7731776
C	-3.0355497	-1.3371939	0.0638357
H	-2.6416318	-1.7601373	0.9941571
C	-3.9636547	-0.2341684	-2.2430629
H	-4.2925472	0.2496811	-3.1667534
C	-0.1913004	-1.9065624	4.6807007
H	-0.4356861	-1.0505679	5.3155226
C	0.3097013	-2.6746341	1.0114704
H	0.5808097	-3.6135726	0.4965232
C	2.8621236	1.6652514	0.3158740
H	2.2862189	2.4087859	-0.2453137
C	4.2494066	1.7096952	0.3173450
H	4.7632994	2.4909887	-0.2491181
C	0.4368518	-4.0667678	3.0264339
H	0.6835083	-4.9072349	2.3685640
C	1.0977060	-0.6506201	-1.7228824
H	2.0916891	-0.6411187	-1.2413999
H	1.2499148	-0.8216622	-2.8039302
C	0.3836593	1.4361935	-2.4698932
H	0.8187463	1.1113675	-3.4319363
C	-0.1965529	2.7610151	-2.5040767
C	0.3545306	-4.2581862	4.3966321
H	0.5342174	-5.2452177	4.8309179
C	0.2073707	-2.8004967	2.4490923
C	0.0368797	-3.1649069	5.2200591
H	-0.0310963	-3.3046599	6.3039403
C	-0.6764671	4.7572453	-3.8172888
H	-0.6249228	5.3064377	-4.7611358
C	4.2296387	-0.2131266	1.7306745
H	4.7268058	-0.9937437	2.3128605
C	6.4686949	0.7783579	1.0997199
H	6.8987255	1.2557953	0.2047035
H	6.8836237	-0.2374600	1.2019854
H	6.7991984	1.3608348	1.9796252
C	4.9732839	0.7527432	1.0413706
C	-0.1391997	3.4838683	-3.7139673
H	0.3422240	3.0190814	-4.5812362
C	2.8419772	-0.1856149	1.6713932
H	2.2495535	-0.9353439	2.2033006
C	-2.8985316	0.3049391	-1.5322862
H	-2.3937144	1.2066323	-1.8901416
C	-4.1027839	-1.9437419	-0.5838958
H	-4.5450499	-2.8493798	-0.1600649
C	0.2823217	-1.7890699	-1.1418996
H	-0.7169883	-1.7891874	-1.6125763
H	0.7680851	-2.7511978	-1.3858584
C	-5.7653124	-1.9915049	-2.4912714
H	-6.7035714	-1.5295320	-2.1321210
H	-5.8375820	-3.0753641	-2.3066535
H	-5.7039839	-1.8132813	-3.5770471
